# Quantifying the Health Burden of COVID-19 Using Individual Estimates of Years of Life Lost Based on Population-wide Administrative Level Data

**DOI:** 10.1097/EDE.0000000000001854

**Published:** 2025-04-09

**Authors:** Elena Milkovska, Bram Wouterse, Jawa Issa, Pieter van Baal

**Affiliations:** From the a Erasmus School of Health Policy and Management, Erasmus University, Rotterdam, The Netherlands.

**Keywords:** Age distribution, COVID-19, Health distribution, Income distribution, Mortality, Years of life lost

## Abstract

**Background:**

The coronavirus disease 2019 (COVID-19) pandemic caused substantial health losses but not much is known about how these are distributed across the population. We aimed to estimate the distribution of years of life lost (YLL) due to COVID-19 and investigate its variation across the Dutch population, taking into account preexisting differences in health.

**Methods:**

We used linked administrative data covering the entire 50+ Dutch population over 2012–2018 (n = 6,102,334) to estimate counterfactual individual-level life expectancy for those who died from COVID-19 in 2020 and 2021. We estimated survival models and used Cox-LASSO and Cox-Elastic Net to perform variable selection among the large set of potential predictors in our data. Using individual-level life expectancy predictions, we generated the distribution of YLL due to COVID-19 for the entire 50+ population by age and income.

**Results:**

On average, we estimate that individuals who died of COVID-19 had a counterfactual life expectancy about 28% lower than that of the rest of the population. Within this average, there was substantial heterogeneity, with 20% of all individuals who died of COVID-19 having an estimated life expectancy exceeding that of the age-specific population average. Both the richest and poorest COVID-19 decedents lost the same average number of YLL, which were similarly dispersed.

**Conclusion:**

Accounting for preexisting health problems is crucial when estimating YLL due to COVID-19. While average life expectancy among COVID-19 decedents was substantially lower than for the rest of the population, the popular notion that only the frail died from COVID-19 is not true.

The coronavirus disease 2019 (COVID-19) pandemic has led to many deaths worldwide.^1^ The health burden associated with those deaths is often quantified using years of life lost (YLL): the remaining years individuals who died of COVID-19 could otherwise have been expected to live. Good estimates of the YLL among COVID-19 decedents are crucial to better understand the consequences of the COVID-19 pandemic, and are relevant for policymakers who have to trade off the health benefits of preventive measures against their societal costs. For this purpose, it is not sufficient to solely quantify total or average YLL; insight into the distribution of these health losses within the population is needed as well. Inequalities in exposure, preexisting health, vaccination uptake, and healthcare access resulted in substantial differences in the risk of COVID-19 death within populations.^2–5^ While the exposure to the virus was largely determined by factors unrelated to health, such as education, type of work, and household composition,^6,7^ the risk of dying conditional on infection was strongly related to preexisting health,^8,9^ healthcare access, and, after the availability of vaccinations, vaccination uptake. The complex interaction between a diverse set of health-related risk factors and societal determinants suggests that there is likely large heterogeneity in the health burden of COVID-19 deaths across but also within different socioeconomic groups.

The standard approach to measure YLL is not suited to quantify this variation in the health burden. In YLL studies, life expectancy is commonly estimated using a population-based lifetable and, consequently, only measures the average years that a particular age-sex group is expected to live. Moreover, it implicitly assumes that those who died of a particular disease had the same life expectancy as the general population conditional on age and sex.^10^ However, because COVID-19 mortality is associated with a substantial number of chronic diseases and risk factors, the remaining life expectancy of those who died of COVID-19 is likely much lower than that of the general population. The handful of studies^11–14^ that have attempted to account for preexisting health problems all find substantial reductions, between 1.5 and 3.5 years, in the estimated average YLL due to COVID-19 mortality compared with the standard approach. Although these studies have different empirical approaches, they all rely on the stratification of their sample of COVID-19 decedents based on a limited set of comorbidities or other health-related variables. For each stratum, life expectancies by age are either obtained from existing studies or estimated. While these studies provide valuable insights into the average YLL for COVID-19 decedents, they cannot provide information on the full underlying distribution, as the number of strata included is naturally limited and the variation within each subgroup remains unobserved.

In this paper, we quantify the full distribution of YLL due to COVID-19 in the Netherlands during 2020 and 2021. We do this by first estimating survival models on the entire Dutch 50+ population in the years prior to the pandemic. We use administrative data with a wide range of indicators of health and apply different data-driven variable selection methods to construct parsimonious survival models. Then, we use these models to produce individual-level predictions of YLL for each COVID-19 decedent in the Netherlands. We visualize the distribution of YLL by age, estimate averages and distributional parameters for COVID-19 decedents and compare these to the rest of the population. Finally, we explore to what extent the heterogeneity in YLL among COVID-19 decedents can be attributed to income.

## METHODS

### Study Design

We estimated the distribution of YLL due to COVID-19 in the Dutch population in several steps. First, we estimated Cox survival models for all-cause mortality over a 7-year follow-up (2012–2018) based on predictors assessed at the start of 2012. Second, we predicted remaining life expectancy for each individual in the entire 50+ population at the start of 2020. We did this by applying the estimated coefficients from the first step to the predictor values at the start of 2020, in combination with extrapolating the baseline survival rates. Third, we identified individuals who died of COVID-19 in 2020 and 2021 and calculated the YLL due to COVID-19 for these individuals as equal to their predicted life expectancy. Fourth, we compared the distribution of predicted life expectancy among COVID-19 decedents to the rest of the population and across income groups.

### Source Data

Statistics Netherlands provided individual-level data on disposable income, personal characteristics, healthcare use, and cause and date of death, linked via pseudonymized personal identification numbers. We obtained personal characteristics, including age and sex, from the Dutch municipality registry. Annual disposable household income, adjusted for household size using an equivalence scale, was sourced from the tax registry. Income was grouped into deciles by age and sex, ensuring equal observations across all deciles for each age-sex group.

To proxy preexisting health status, we used outpatient medication prescriptions, nursing home admissions, functional impairments, and hospitalizations. Annual data on medication prescriptions under Dutch mandatory social health insurance were classified by the anatomical therapeutic chemical (ATC) 2nd level (therapeutic subgroup),^15^ resulting in 87 medication groups. Data from the Care Assessment Centre created an indicator for functional impairment (somatic, psychogeriatric, psychiatric, physical, or any other mental incapacitation), scored as one if eligible for care. The central administration office provided nursing home use data, indicating if an individual used such care in the previous year. The National Basic Register of Hospital Care (Landelijke Basisregistratie Ziekenhuiszorg/Landelijke Medische Registratie)^16^ included almost all inhospital admissions, classified by the ICD 9th and 10th editions^17^ into 18 groups based on the International Shortlist for Hospital Morbidity Tabulation.^18^ Variables indicated urgency, stay duration, and number of admissions per diagnosis group. Pregnancy-related hospitalizations were excluded.

The Dutch cause of death registry provided (main) underlying cause of death (classified via ICD-10) and date of death. We used codes U07.1 (confirmed COVID-19) and U07.2 (suspected COVID-19) to identify COVID-19-related deaths.^19^

The project has received approval for data access and results dissemination from the Centraal Bureau voor de Statistiek (Statistics Netherlands). No further ethical approval is required.

### Statistical Analysis

#### Estimating Survival Models

We estimated Cox proportional hazards models using all-cause mortality for the entire 50+ population over the span of 7 years: 2012–2018, using predictors from the start of 2012. The data are recent but also allowed for a sufficiently long enough follow-up to estimate survival models. The analysis was restricted to those 50 or older, as more than 99% of COVID-19 deaths occurred in that age group. We performed stratified analyses by income, sex, and nursing home usage, as we expected the baseline hazard to differ strongly between these groups but also to avoid the models potentially favoring certain subgroups. As the majority of Dutch nursing home admissions are permanent,^20^ we stratified based on whether individuals were in a nursing home at the start of 2012. We, thus, used a total of 40 regressions: men and women, in and out of nursing homes, for each income decile.

The considered predictors were age at baseline, outpatient medication use at therapeutic group level (87 in total), functional impairment at the type of care level (five in total), number/urgency/duration of hospital admissions at International Shortlist for Hospital Morbidity Tabulation–group level (18 diagnoses levels, and 72 variables in total) (eAppendix S3; http://links.lww.com/EDE/C229). Age was a categorical variable by 1-year group for non-nursing home dwellers. Because stratifying by income and sex led to fewer events in richer nursing home residents, we grouped their age in 5-year groups to avoid failure to converge or unrealistically high coefficients due to zero deaths in certain predictor combinations. We included outpatient medication use in the regressions for the non-nursing home groups only. We also considered the care intensity package of nursing home admissions (10 in total). Age and functional impairment were based on the values on 1 January 2012, whereas the other variables were lagged annual values based on 2011.

The data provided a large number of potential predictors (over 200). To prevent overfitting, we used LASSO and Elastic Net regularization with five-fold cross-validation.^21^ Regular Cox proportional hazards regressions were also estimated with and without accounting for hospitalization urgency and stay. We evaluated out-of-sample model performance on the 2019 population using Harrell’s C-index^22^ and calibration metrics.^23^ eAppendix S3; http://links.lww.com/EDE/C229 describes what constitutes good model performance metrics and presents them in full. Based on the out-of-sample performance, we selected the preferred estimates to use for the prediction of the hazard ratio for all-cause mortality. Results by income are presented in eAppendix S4; http://links.lww.com/EDE/C229.

Finally, because the Cox model does not impose a functional form on the baseline hazard rate, we followed Deryugina et al.^24^ and estimated a seasonally corrected log-linear relation between the baseline hazard and time. The resulting baseline hazard was extrapolated for an additional 43 years and converted to a baseline survival curve.

#### Individual Predictions of Life Expectancy

Using the preferred estimation model, we predicted the hazard ratio for all-cause mortality for each individual in the entire 50+ population at the start of 2020. We made these predictions using the estimated coefficients from the previous step and applying these to the values of the selected predictors at the start of 2020. Next, we combined the all-cause hazard ratio and the extrapolated baseline hazard. The outcome was an individual-level survival curve, which once integrated over time resulted in an individual-level remaining life expectancy for each person of the Dutch 50+ population.

We used the death records for 2020 and 2021 to identify individuals who died from COVID-19. We equated the YLL due to COVID-19 for these individuals to their remaining LE. We plotted the resulting distribution of life expectancy (YLL) across all individuals who died of COVID-19 and for the rest of the Dutch population. We reported the following statistics: the mean, median, and the quartile coefficient of variation as a measure of spread. We also reported similar outcomes between income groups and used an analysis of variance decomposition to determine to what extent do income deciles explain the variation in remaining life expectancy. To assure privacy while working with the administrative data, but also for ease of presentation, results are grouped into income quintiles instead of deciles, where for example, the bottom/top quintile combines the bottom/top two deciles.

## RESULTS

First, we discuss the selection of the survival model used to predict life expectancy. This is followed by a comparison of the individual-level life expectancy distributions between COVID-19 decedents and everyone else, also considering income. Table 1 shows a truncated overview of the population characteristics, with a full version available in eTables S1–S7; http://links.lww.com/EDE/C229.

**TABLE 1.** Descriptive Statistics by Income for the Population Aged 50+ in the Sample Used for Estimating the Survival Models (2012–2018), and the Sample Used for Generating Results (2020–2021)

Estimation Sample (2012-2018), Population Aged 50+				
	D1 (Poorest)	D2	D9	D10 (Richest)
	N = 610,383	N = 610,278	N = 610,198	N = 610,163
Average age, years (Mean [SD])	64.7 (10.6)	64.7 (10.6)	64.7 (10.6)	64.7 (10.6)
All-cause deaths over the course of follow-up	133,674 (22%)	115,343 (19%)	79,326 (13%)	76,270 (13%)
Lost to follow-up	12,208 (2%)	5,493 (1%)	3,051 (1%)	5,491 (1%)
Are male	290,542 (48%)	290,492 (48%)	289,844 (48%)	289,827 (48%)
Presence of any functional impairment	137,336 (23%)	89,101 (15%)	40,883 (7%)	37,220 (6%)
In a nursing home	77,519 (13%)	32,955 (5%)	14,035 (2%)	12,813 (2%)
Have used any medication^a^	449,375 (84%)	497,774 (86%)	489,320 (82%)	483,213 (81%)
Have been hospitalized for any reason^b^	96,441 (16%)	103,137 (17%)	86,648 (14%)	83,592 (14%)
**Prediction Sample (2020-2021), Population Aged 50+**				
	**D1 (Poorest**)	**D2**	**D9**	**D10 (Richest**)
	**N = 702,948**	**N = 702,832**	**N = 702,778**	**N = 702,727**
Average age, years (Mean [SD])	65.4 (10.7)	65.4 (10.7)	65.4 (10.7)	65.4 (10.7)
All-cause deaths over the course of follow-up	51,315 (7%)	42,170 (6%)	24,597 (4%)	23,893 (3%)
Lost to follow-up	11,950 (2%)	11,245 (2%)	12,650 (2%)	12,649 (2%)
COVID-19 deaths over the course of follow-up	7,281 (1%)	5,497 (1%)	2,507 (0%)	2,299 (0%)
Are male	338,821 (48%)	338,765 (48%)	338,739 (48%)	338,714 (48%)
Presence of any functional impairment	75,215 (11%)	33,736 (5%)	12,650 (2%)	11,946 (2%)
In a nursing home	63,265 (9%)	23,193 (3%)	7,731 (1%)	7,027 (1%)
Have used any medication^a^	539,485 (84%)	578,323 (85%)	558,966 (80%)	550,523 (79%)
Have been hospitalized for any reason^b^	111,769 (16%)	116,670 (17%)	92,767 (13%)	89,246 (13%)

Each column presents the population grouped into sex- and age-specific income deciles. D1, D2, D9, and D10 denote sex- and age-specific income deciles.

a This was generated for descriptive purposes only. The analysis uses detailed information on medication usage based on anatomical therapeutic chemical 2nd-level classification.

b For descriptive purposes only. The analysis uses detailed data on hospitalizations by diagnoses, duration of admission, and urgency.

SD indicates standard deviation.

### Model Selection

All survival models had decent discrimination capability and calibration, and none of the models were clearly superior to the others. In the end, we chose the Elastic Net as our main analysis because it had the highest validation C-index—an average of 0.75 across all stratified analyses. Also, its calibration slope was the closest to one (an average of 0.99) and its calibration intercept was indistinguishable from zero across the most groups. eTables S9 and S10; http://links.lww.com/EDE/C229 present the performance measures for the models in more detail.

### Life Expectancy for COVID-19 Decedents and the Rest of the Population

Estimates of life expectancy indicated that individuals who died of COVID-19 were on average less healthy than the rest of the population. The difference in average life expectancy between COVID-19 decedents and the rest of the population ranged from −4.3 (−22%) for a 60-year-old and −2.1 years (−32%) for an 85-year-old, which on average results in a difference of −2.5 years (−28%) across all COVID-19 deaths. The crude difference in average life expectancy, irrespective of age and how COVID-19 mortality is distributed, is −10.1 years (−61%) (eAppendix S4; http://links.lww.com/EDE/C229). The average YLL among all COVID-19 decedents is 6.5, which is 2.5 less compared with an estimate using the standard life table approach (9.0) (Table 2).

**TABLE 2.** COVID-19 Deaths, Average Age-at-death and Average Years of Life Lost for the Population of the Netherlands Who Were Aged 50+ at the Start of 2020 and Deceased due to COVID-19 in the Span of 2020–2021, by Income

2020–2021 Combined						
		Number of COVID-19 Deaths^a^	Age-at-death^b^	Average Years of Life Lost^a^		
				Standard Life Table^c^	Income-stratified Life Table^c^	Individual-level LE Method^d^
All COVID-19 decedents	D1&D2 (poorest)	12,778 (33%)	79.6	9.8 (36%)	8.5 (32%)	6.4 (32%)
	D3&D4	8,615 (22%)	80.5	9.2 (23%)	9.1 (23%)	6.8 (23%)
	D5&D6	7,060 (18%)	81.5	8.6 (17%)	8.9 (18%)	6.6 (18%)
	D7&D8	6,004 (15 %)	82.1	8.3 (14%)	8.8 (15%)	6.7 (16%)
	D9&D10 (richest)	4,806 (12%)	83.1	7.7 (11%)	8.4 (12%)	6.3 (12%)
	All income groups	39,263 (100%)	81.0	9.0 (100%)	8.7 (100%)	6.5 (100%)

Income deciles were grouped into quintiles.

a Values in brackets are percentages of the total deaths/years of life lost burden.

b Age-at-death is as of 1 January 2020.

c Income was taken as income deciles; 2019 population 50+ data stratified by sex was used for the life tables.

d Individual-level life expectancy is as of 1 January 2020 for all decedents.

**FIGURE 1.**
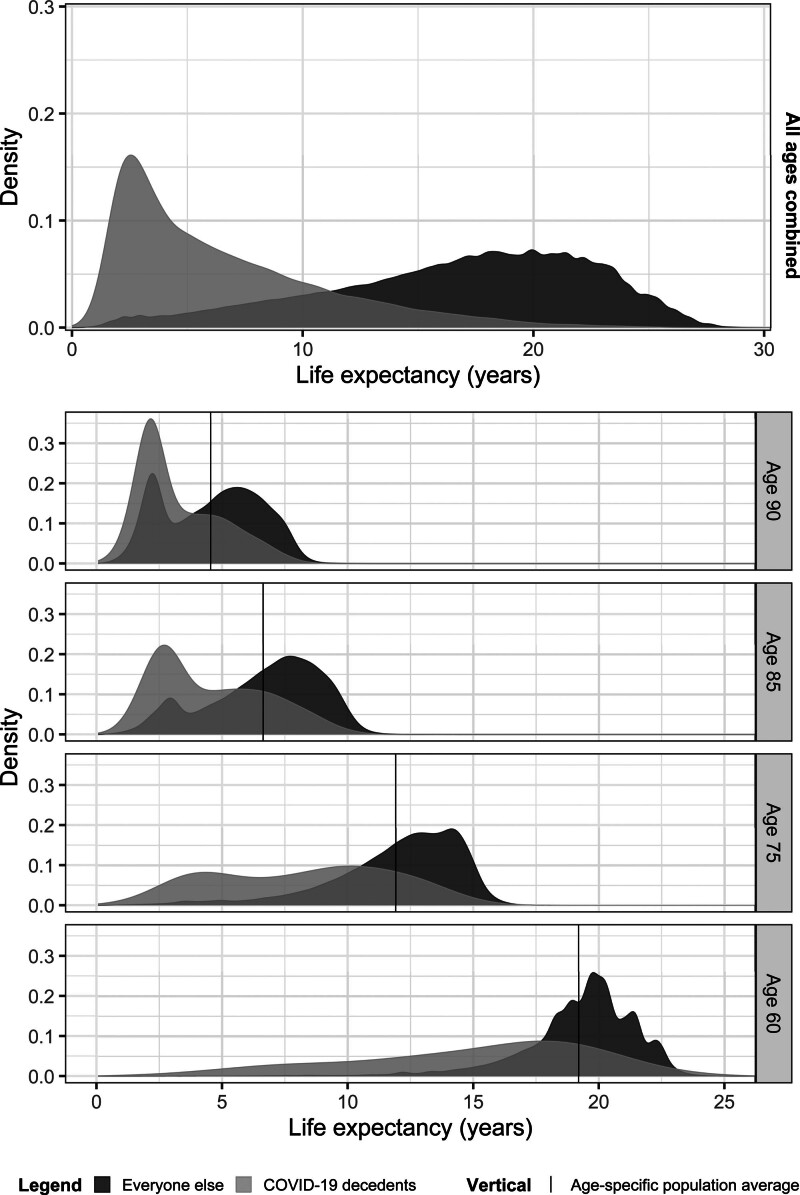
Distribution of predicted individual-level remaining life expectancies of those who died from COVID-19 (i.e., YLL) in the Netherlands 2020–2021 (in gray) versus everyone else (dark gray)—total and at selected ages. The plot displays the density, that is, the area under the curve equals one. The distributions include both men and women who were aged 50+ as of 1 January 2020.

There was considerable individual heterogeneity underlying these averages. Figure 1 depicts the distribution of life expectancy in COVID-19 decedents and the rest of the population for the entire 50+ population and by selected age groups. The distribution of the life expectancy of the COVID-19 decedents is clearly skewed toward the left compared with that of the rest of the population (see eAppendix S4; http://links.lww.com/EDE/C229 for distributional metrics). At the same time, the distributions for COVID-19 decedents overlap with those of the rest of the population across all life expectancy values and 20% of the people who died of COVID-19 actually had a life expectancy higher than the age-specific average.

### Years of Life Lost by Income

**FIGURE 2.**
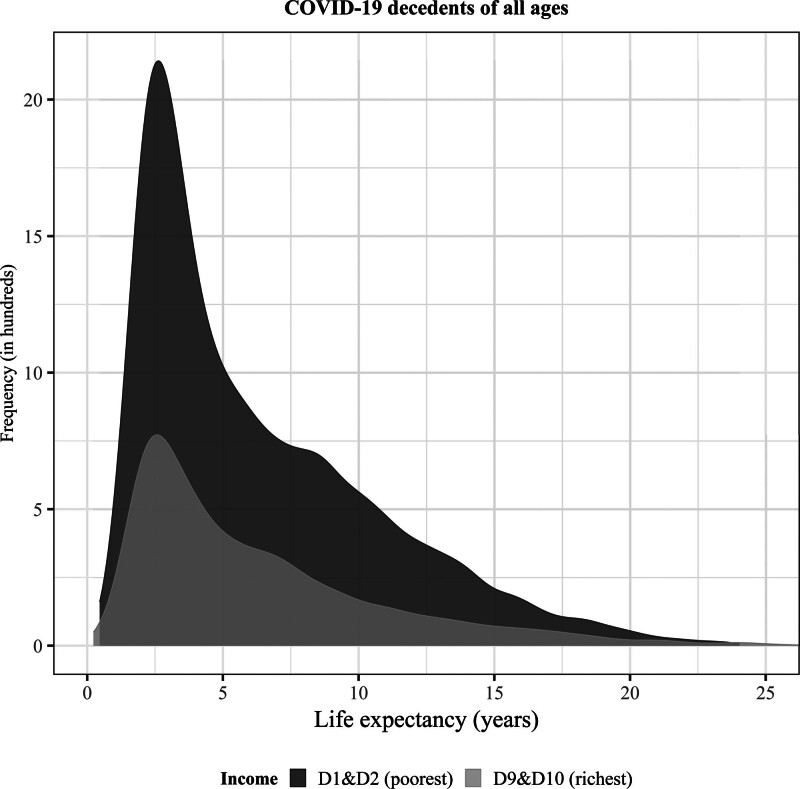
Distribution of predicted life expectancy for Dutch men and women aged 50+ of 1 January 2020 who died of COVID-19 in the span of 2020–2021, by income quintiles. D1&D2 = first two income deciles, poorest; D9&D10 = last two income deciles, richest. Tails have been cut off at 0.05% for the sake of better presentation. The plot displays the frequency of the values in hundreds, that is, the density corrected for the number of people.

**FIGURE 3.**
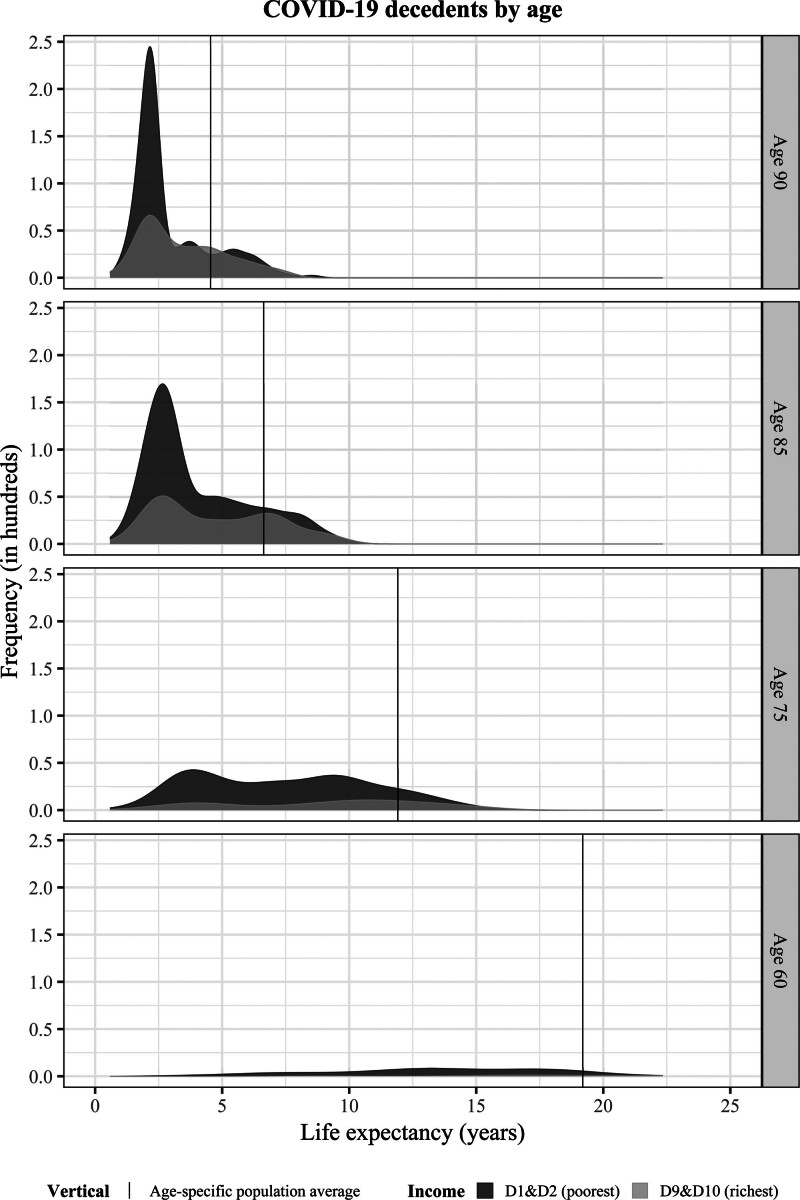
Distribution of predicted life expectancy for Dutch men and women across selected ages who died of COVID-19 in the span of 2020–2021, by income quintiles. D1&D2 = first two income deciles, poorest; D9&D10 = last two income deciles, richest. Tails have been cut off at 0.05% for the sake of better presentation. The plot displays the frequency of the values in hundreds, that is, the density corrected for the number of people.

Figure 2 (as well as Table 2) shows that the number of COVID-19 deaths is 2.7 times higher in the lowest two income deciles than in the highest: 12,778 individuals in the lowest income group died of COVID-19 compared with 4,806 in the highest. That increased mortality for the poor persisted when looking separately at 2020 (when no vaccination was available) and 2021 (when vaccination became available) (eTables S15 and S16; http://links.lww.com/EDE/C229), and distributions by income also have similar medians and interquartile ranges (eTable S14; http://links.lww.com/EDE/C229). Two things stand out. First, despite this difference in the number of deaths, the shape of the distribution of YLL within the two groups seems similar, with the majority of deaths occurring among individuals whose predicted life expectancy is below the age-specific average (Figure 3). This also holds true upon examining by-year results. This suggests that, even when vaccination became available, underlying health remained an important factor in driving COVID-19 mortality. Second, in both years as well as over the whole 2-year period we see comparable patterns across income on three fronts. Average YLL per COVID-decedent are similar—for lowest and highest incomes in 2020 and 2021 although the average age-at-death is a bit lower in all income groups in 2021 compared with 2020 (Table 2 and eTables S15 and S16; http://links.lww.com/EDE/C229). However, the share of total YLL is equally distributed across income groups in 2020 and 2021. These similar results in 2020 and 2021 underscore the relevant role of preexisting health.

For comparison, Table 2 also shows average YLL based on the standard population lifetable approach and on lifetables stratified by income. Average YLL are higher across the board in the standard lifetable approach. Also, they have a strong income gradient, solely driven by the lower average age-at-death for lower incomes. This is showcased by the fact that income-stratified averages lack the income gradient in YLL. Still, these stratified estimates result in much higher estimates of average YLL than our method, suggesting that within income groups those with relatively poor health were more likely to die of COVID-19.

**FIGURE 4.**
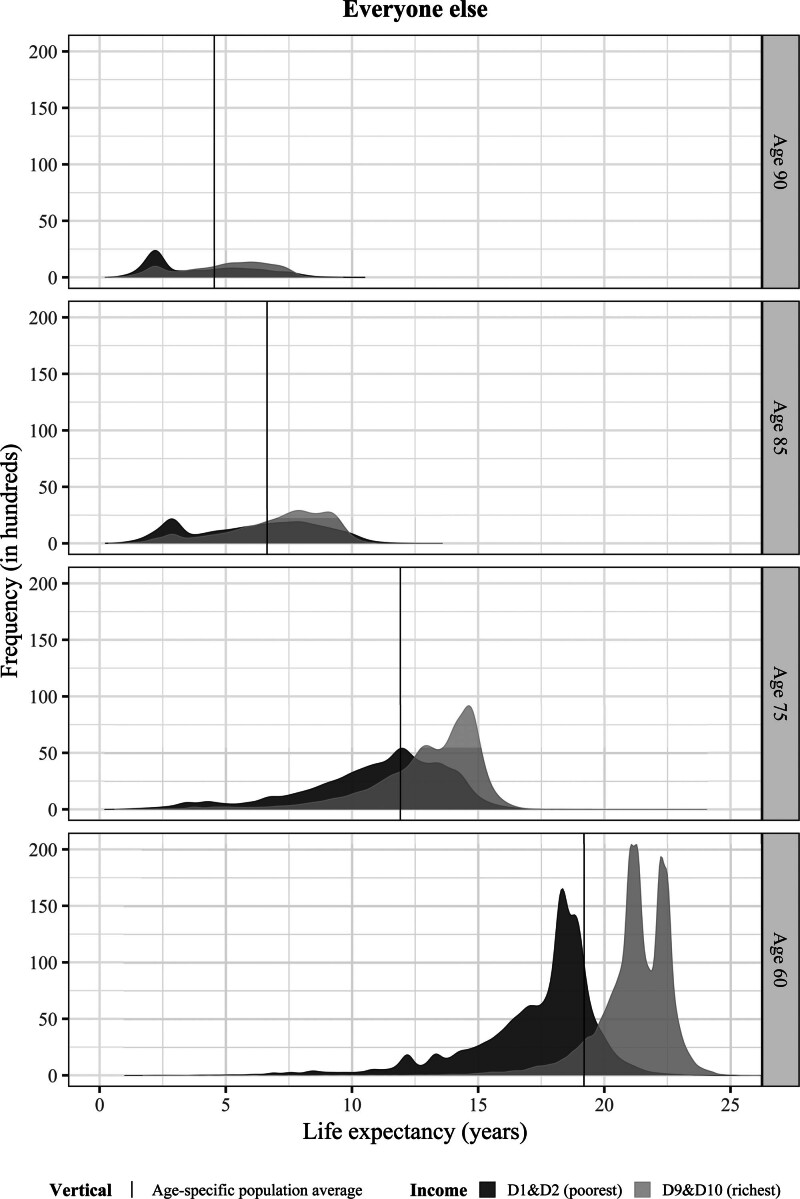
Distribution of predicted life expectancy for Dutch men and women across selected ages who did not die from COVID-19, by income quintiles. D1&D2 = first two income deciles, poorest; D9&D10 = last two income deciles, richest. Tails have been cut off at 0.05% for the sake of better presentation. The plot displays the frequency of the values in hundreds, that is, the density corrected for the number of people.

Figure 3 shows the COVID-19 deaths in the lowest and highest two income deciles by age. Even though the richest decedents were on average in somewhat better health compared with their same-aged low-income counterparts across all ages (Figure S9; http://links.lww.com/EDE/C229), the distribution in YLL by age is quite similar between low and high incomes, especially compared with the much larger differences between low and high incomes in the rest of the population (Figure 4).

Because of the similar distribution of YLL of COVID-19 decedents, income explains little of the variation in YLL among all COVID-19 decedents. In the variance analysis, income explains under 0.3% of the variation of YLL unconditional on age, and an average of almost 6% of the variation by age group. For comparison, income explains 10% of age-specific variation in life expectancy for the rest of the population (eTable S18 in eAppendix S4; http://links.lww.com/EDE/C229 provides detailed life expectancy variance disaggregation).

## DISCUSSION

To our knowledge, our study is the first to produce individual-level estimates of life expectancy and the consequent distribution of YLL due to COVID-19 for a country’s entire population. We did so using an extensive set of health-related predictors and data-driven variable selection methods. Our estimates of average YLL due to COVID-19 confirm the notion that individuals who died of COVID-19 were in considerably worse health than their same-age counterparts in the rest of the population. However, when we go beyond the average the picture becomes more nuanced. We found considerable variation in the YLL among COVID-19 decedents, with 20% of those dying of COVID-19 having a higher life expectancy than the age-specific average. We further found that, while individuals with a lower income are more likely to have died from COVID-19, among those dying of COVID-19 the distributions of YLL were actually very similar across income groups.

### Strengths of the Study

The main strength of this study is the access to extensive administrative data on health, healthcare use, and mortality for the entire Dutch population prior and during the COVID-19 pandemic. As we had comprehensive health and survival information in the years prior to the pandemic, we were able to estimate survival models with a long follow-up based on detailed information on prior health. Consequently, we used these to estimate a (counterfactual) life expectancy for everyone who died during the pandemic and the rest of the population. Our study produced a high-quality estimate of the full distribution of YLL due to COVID-19 mortality in the Netherlands, allowing for detailed subgroup analysis.

### Limitations

Our analysis has several limitations. First, we used administrative healthcare data for the entire population, which may not capture all relevant health aspects influencing mortality.^25,26^ Although our data are comprehensive, the absence of more detailed (e.g., clinical) information is a necessary trade-off for estimating life expectancy across the entire population. For instance, additional insights from data on COVID-19 risk factors like obesity and smoking were not included.^27^ Nevertheless, by including data on medication and hospitalization without identifying the underlying diseases, we let the data speak for itself and determine which is most important for predicting all-cause mortality. Capturing one’s true health state is an inherently difficult task, but even so, healthcare use (and consequently predicted remaining life expectancy) is still an important indicator of health. Second, our estimates, relying on healthcare data, might partially reflect income-based variations in access to care. Given that the Netherlands has one of the most accessible healthcare systems in the world,^28^ we consider this a minor concern. In addition, our analysis explored the heterogeneity in the health burden of COVID-19 deaths of only one dimension of socioeconomic status, that is, income. Data on other dimensions of socioeconomic status were not available for the entire Dutch population and therefore we chose to use only income. Third, we estimated life expectancy at the beginning of 2020, potentially overestimating the actual life expectancy for those COVID-19 decedents who experienced non-COVID-related health deteriorations directly preceding death. Fourth, we identified COVID-19 deaths based on registered underlying causes, dependent on clinician evaluations. While coding practices’ variations are possible, the high-quality COVID-19 death registration in the Netherlands suggests minimal impact.^19^ Finally, our estimates focused solely on YLL due to COVID-19 mortality, excluding non-lethal effects of infection and broader societal responses to the pandemic, such as delayed care.

### Comparison With Other Studies

By having an estimate of the YLL distribution, our study provides an important step to disentangle the close-knit relationship between the burden of COVID-19 and heterogeneity in preexisting health, and income. Previous studies have demonstrated that the average YLL decreases noticeably once the poorer underlying health of COVID-19 decedents is taken into account—from 10.5 to 9.2 for Hungarians,^14^ from 7.5/8.6 to 5.4/6.6 for Swedish women/men,^12^ and from 7.8/8.5 to 4.8/5.3 for Dutch women/men.^13^ Our results align with these findings, as we found the average YLL decreased from 9.0 to 6.5 after controlling for preexisting health. While these other studies relied on estimates of LE by a limited number of subgroups, our analysis leveraged exceptionally detailed data including comprehensive healthcare indicators for both COVID-19 decedents and the overall population, to compute individual-level LEs for the entire Dutch population aged 50+. These estimates showed that while population life expectancy is indeed an overestimate of the average YLL, for 20% of the COVID-19 decedents, it is actually an underestimate.

### The Broader Picture

Our findings are relevant from both a scientific and policy perspective. In public discourse, especially during the early stages of the pandemic, many argued that COVID-19 mortality is selective, and thus, the true burden of disease is lower than standard methods would have us believe. While our findings confirm that accounting for prior health status indeed lowers the estimates of YLL, the disease burden of COVID-19 was still far from negligible, with 6.5 YLL per COVID-19 death. Importantly, the relationship between prior health and COVID-19 mortality was similar across income groups, even though the average age-at-death was lowest among lower income groups. In other words, the average poor younger COVID-19 decedent had similar remaining life expectancy as a rich older one. As such, from a scientific perspective, it is crucial to control for prior health when estimating the income-related inequality in the disease burden of COVID-19.

From a policy perspective, these estimates provide useful information for policymakers who need to weight the costs and benefits of preventive measures across socioeconomic groups during a pandemic. It is important to note that, even when taking health differences into account, the lowest income groups were still estimated to bear the largest share of the disease burden. Moreover, the worse underlying health of low-income groups are the result of structural socioeconomic inequalities in health, meaning that these prior inequalities are likely to shape the health consequences of future pandemics if left unchecked. Another important aspect is that despite the average worse health of individuals who succumbed to COVID-19, a notable share was in good health, with life expectancies equal to or higher than age-specific population averages. This further underscores the need for preventive measures, challenging the misconception that only those in poor health face the risk of COVID-19 mortality. In addition, while the estimation process aims to provide a precise measurement of the impact on health, the interpretation and policy responses may vary. Even so, recognizing that a substantial share of individuals stands to lose many life years is likely to garner increased support for preventive measures. More generally, the finding that there was a large heterogeneity in YLL opens the door for future research to investigate the inner workings of the COVID-19 pandemic.

Interestingly, variation in YLL among COVID-19 decedents was not driven by income even though we found large differences in the probability of COVID-19 death and income has been found to be associated with COVID-19 infection^7,29^ and vaccination uptake.^4^ The equal distribution of YLL suggests that the richest and poorest COVID-19 decedents were in the same general health prior to their death and that the main driver of income inequality in total YLL is the number of deaths. We posit that this intriguing result is at least partly due to the equal healthcare access in the Netherlands. For countries with less equitable access to healthcare, we might expect more right-skewed distributions of YLL in low-income groups, as lack of access to proper COVID-care likely results in more low-income individuals with relatively good health dying of COVID-19.

While our research cannot draw direct conclusions about the role of exposure and vaccination uptake, our findings underline the key role of preexisting health and the need for public health policy to decrease the income inequalities in health in general. Both in 2020 and 2021, we see resemblances in the patterns cross income groups when it comes to average YLL per COVID-decedent (similar across income groups), average age of death (lower among lower income groups), and the distribution of the total YLL (highly concentrated among the lowest income groups). This suggests that also in 2021, when vaccinations became available, differences in underlying health still played an important role in the distribution of the disease burden of COVID-19 mortality across income groups. These differences in underlying health can at least partially be attributed to the fact that chronic diseases are not equally spread across the population and are instead centered in people with lower socioeconomic status.^30^ Therefore, addressing general income inequalities in health appears to be an essential step for public policy if we aim to be better prepared for future pandemics. However, more research is warranted to disentangle the income-related health burden of COVID-19, given that income has differential effects on the three crucial determinants of COVID-19 mortality (exposure, prior health, and vaccination uptake).

## CONCLUSION

Although preexisting health had a large impact on estimates of YLL, there was a large degree of variation in YLL across the Dutch population indicating that not only the frail and sick died of COVID-19. Similarities in the distributions of YLL of different income groups suggest that preexisting income-related health inequalities have played an important part in explaining the health burden of COVID-19 even in a country with equal access to healthcare.

## Supplementary Material

## References

[1] Wang H Paulson KR Pease SA. Estimating excess mortality due to the COVID-19 pandemic: a systematic analysis of COVID-19-related mortality, 2020-21. Lancet. 2022;399:1513–1536. 35279232 10.1016/S0140-6736(21)02796-3 PMC8912932

[2] Nordberg P Jonsson M Hollenberg J. Immigrant background and socioeconomic status are associated with severe COVID-19 requiring intensive care. Sci Rep. 2022;12:1–10. 35840691 10.1038/s41598-022-15884-2 PMC9285186

[3] Stoeldraijer L Kunst A Chilunga F Harmsen C. Sociaal-demografische verschillen in COVID-19-sterfte in het eerste jaar van de coronapandemie. 2022. Available at: https://www.cbs.nl/nl-nl/longread/statistische-trends/2022/sociaal-demografische-verschillen-in-covid-19-sterfte-in-het-eerste-jaar-van-de-coronapandemie. Accessed 12 April 2023.

[4] Pijpers J van Roon A van Roekel C. Determinants of COVID-19 vaccine uptake in The Netherlands: a Nationwide Registry-Based Study. Vaccines (Basel). 2023;11:1409. 37766087 10.3390/vaccines11091409 PMC10537724

[5] Smolić S Čipin I Međimurec P. Access to healthcare for people aged 50+ in Europe during the COVID-19 outbreak. Eur J Ageing. 2021;19:793–809. 34149338 10.1007/s10433-021-00631-9 PMC8195455

[6] Pagen DME Brinkhues S Dukers-Muijrers NHTM. Exposure factors associated with SARS-CoV-2 seroprevalence during the first eight months of the COVID-19 pandemic in the Netherlands: a cross-sectional study. PLoS One. 2022;17:e0268057. 35551285 10.1371/journal.pone.0268057 PMC9097988

[7] Vos ERA Van Boven M Den Hartog G. Associations between measures of social distancing and severe acute respiratory syndrome coronavirus 2 seropositivity: a nationwide population-based study in the Netherlands. Clin Infect Dis. 2021;73:2318–2321. 33772265 10.1093/cid/ciab264 PMC8083720

[8] Gao Y Ding M Dong X. Risk factors for severe and critically ill COVID-19 patients: a review. Allergy. 2021;76:428–455. 33185910 10.1111/all.14657

[9] Nijman G Wientjes M Ramjith J. Risk factors for in-hospital mortality in laboratory-confirmed COVID-19 patients in the Netherlands: a competing risk survival analysis. PLoS One. 2021;16:e0249231. 33770140 10.1371/journal.pone.0249231 PMC7997038

[10] Aburto JM Schöley J Kashnitsky I. Quantifying impacts of the COVID-19 pandemic through life-expectancy losses: a population-level study of 29 countries. Int J Epidemiol. 2022;51:63–74. 34564730 10.1093/ije/dyab207 PMC8500096

[11] Wouterse B Ram F van Baal P. Quality-adjusted life-years lost due to COVID-19 mortality: methods and application for The Netherlands. Value Health. 2022;25:731–735. 35500946 10.1016/j.jval.2021.12.008 PMC8810280

[12] Ebeling M Acosta E Caswell H Meyer AC Modig K. Years of life lost during the Covid-19 pandemic in Sweden considering variation in life expectancy by level of geriatric care. Eur J Epidemiol. 2022;37:1025–1034. 36127511 10.1007/s10654-022-00915-z PMC9488891

[13] Issa J Wouterse B Milkovska E van Baal P. Quantifying income inequality in years of life lost to COVID-19: a prediction model approach using Dutch administrative data. Int J Epidemiol. 2023;53:dyad159. 10.1093/ije/dyad159 PMC10859130 38081182

[14] Ferenci T. Different approaches to quantify years of life lost from COVID-19. Eur J Epidemiol. 2021;36:589–597. 34114188 10.1007/s10654-021-00774-0 PMC8192042

[15] World Health Organization (WHO). ATC classification index with DDDs. WHO Collaborating Centre for Drug Statistics Methodology. 2023. Available at: https://www.whocc.no/atc/. Accessed 6 October 2023.

[16] CBS-Microdata. Documentatierapport Ziekenhuisopnamen Landelijke Basisregistratie Ziekenhuiszorg (LBZBASISTAB). Available at: https://www.cbs.nl/nl-nl/onze-diensten/maatwerk-en-microdata/microdata-zelf-onderzoek-doen/microdatabestanden/lbzbasistab-ziekenhuisopnamen-lbz. Accessed 6 October 2023.

[17] World Health Organization (WHO). International classification of diseases, tenth revision (ICD-10). 2019. Available at: https://icd.who.int/browse10/2019/en. Accessed 6 October 2023.

[18] International shortlist for hospital morbidity tabulation (ISHMT). https://stats.oecd.org/wbos/fileview2.aspx?IDFile=e477970b-3024-4188-8dc6-13f3db201846. Accessed 27 June 2023.

[19] Statistics Netherlands. Adviezen CBS gebruik van COVID-19 op doodsoorzaakverklaring (B-formulier). 2020. Available at https://www.cbs.nl/nl-nl/deelnemers-enquetes/decentrale-overheden/decentrale-overheid/doodsoorzaak/adviezen-cbs-gebruik-van-covid-19-op-doodsoorzaakverklaring--b-formulier--. Accessed 6 October 2023.

[20] Wouterse B Bakx P Wong A. Measuring nursing home performance using administrative data. Med Care Res Rev. 2023;80:187–204. 35872642 10.1177/10775587221108247 PMC10009495

[21] James G Witten D Hastie T Tibshirani R. An introduction to statistical learning. Springer; 2013:112.

[22] Newson RB. Comparing the predictive powers of survival models using Harrell’s C or Somers’ D. Stata J. 2010;10:339–358.

[23] Royston P. Tools for checking calibration of a Cox model in external validation: approach based on individual event probabilities. Stata J. 2014;14:738–755.

[24] Deryugina T Heutel G Miller NH Molitor D Reif J. The mortality and medical costs of air pollution: evidence from changes in wind direction. Am Econ Rev. 2019;109:4178–4219. 32189719 10.1257/aer.20180279 PMC7080189

[25] Bohensky MA Jolley D Pilcher DV Sundararajan V Evans S Brand CA. Prognostic models based on administrative data alone inadequately predict the survival outcomes for critically ill patients at 180 days post-hospital discharge. J Crit Care. 2012;27:422.e11–422.e21. 10.1016/j.jcrc.2012.03.008 22591572

[26] Andrew Lin CH Usn M John Chin LC Sicignano NM Evans AM. Repeat hospitalizations predict mortality in patients with heart failure. Mil Med. 2017;182:1932. 10.7205/MILMED-D-17-00017 28885958

[27] Mahamat-Saleh Y Fiolet T Rebeaud ME. Diabetes, hypertension, body mass index, smoking and COVID-19-related mortality: a systematic review and meta-analysis of observational studies. BMJ Open. 2021;11:e052777. 10.1136/bmjopen-2021-052777 PMC8557249 34697120

[28] Schneider EC Shah A Doty MM. Reflecting poorly: health care in the US compared to other high-income countries. The Commonwealth Fund, 4; 2021. Available at: https://www.commonwealthfund.org/sites/default/files/2021-08/Schneider_Mirror_Mirror_2021.pdf.

[29] Upshaw TL Brown C Smith R Perri M Ziegler C Pinto AD. Social determinants of COVID-19 incidence and outcomes: a rapid review. PLoS One. 2021;16:e0248336. 33788848 10.1371/journal.pone.0248336 PMC8011781

[30] Mackenbach JP Stirbu I Roskam AJR; European Union Working Group on Socioeconomic Inequalities in Health. Socioeconomic inequalities in health in 22 European Countries. N Engl J Med. 2008;358:2468–2481. 18525043 10.1056/NEJMsa0707519

