# Supplementary material for: Quantifying the Health Burden of COVID-19 Using Individual Estimates of Years of Life Lost Based on Population-wide Administrative Level Data
Source: Epidemiology. 2025 Apr 9;36(4):520–30. doi: 10.1097/EDE.0000000000001854 (PMC12118614; doi:10.1097/EDE.0000000000001854)
Supplement: Supplementary file 1 [file ede-36-520-s001.pdf]

Quantifying the health burden of COVID-19 using individual estimates of years of life lost based on population-wide administrative level data

Elena Milkovska[1], Bram Wouterse[1], Jawa Issa[1], Pieter van Baal[1]

[1] Department of Health Economics, Erasmus School of Health Policy and Management, Erasmus University Rotterdam, Rotterdam, The Netherlands

APPENDIX

Contents

S1. Description of different samples 3
S2. Population characteristics 4
S3. Detailed methodology of life expectancy prediction 12
Study design 12
Model estimation 12
Regular Cox model 14
Regularization models 15
Model performance and external validation 16
Discrimination 16
Calibration 17
Baseline hazard function and extrapolation 21
Elastic Net standardized coefficients 22
S4. More life expectancy results 35
References 43

Table S1 Created datasets used for analysis, validation, and results. 3
Table S2 Population characteristics by income deciles for the estimation and prediction samples 4
Table S3 Hospitalizations in 2011 by diagnosis and income for the people in the Baseline sample. 6
Table S4 Hospitalizations in 2019 by diagnosis and income for the people in the Prediction sample who did not die from COVID-19. 6

Table S5 Hospitalizations in 2019 by diagnosis and income for the people in the Prediction sample who died from COVID-19. 8
Table S6 Medication usage by income across different samples – Women. 9
Table S7 Medication usage by income across different samples – Men. 10
Table S8 Types of regression and analyses. 13
Table S9 Ability or each model to discriminate observations with/without the event. 17
Table S10 Calibration slope averaged out across income deciles for ease of presentation. 20
Table S11 Variables that were selected by the Elastic Net for the final analysis, non-nursinghome 23
Table S12 Variables that were selected by the Elastic Net for the final analysis, nursinghomes. 27
Table S13 Predicted life expectancy distribution characteristics across all income groups, 2020-2021. 35
Table S14 Predicted life expectancy distribution characteristics across by income.. 36
Table S15 COVID-19 deaths, average age-at-death and average years of life lost for the population of the Netherlands who were aged 50+ at the start of 2020 and deceased due to COVID-19 in the span of 2020, by sex and income quintile. 40
Table S16 COVID-19 deaths, average age-at-death and average years of life lost for the population of the Netherlands who were aged 50+ at the start of 2020 and deceased due to COVID-19 in the span of 2021, by sex and income quintile. 41
Table S17 COVID-19 deaths, average age-at-death and average years of life lost for the population of the Netherlands who were aged 50+ at the start of 2020 and deceased due to COVID-19 in the span of 2020-2021, by sex and income quintile, by sex and income quintile... 42
Table S18 ANOVA disaggregation of the life expectancy variation. 42

Figure S1 Baseline population size for each stratified survival analysis. 4
Figure S2 Natural logarithm of the baseline hazard function across time 22
Figure S3 Standardized age coefficients for people not in nursing homes from the chosen model. 29
Figure S4 Standardized age coefficients for people in nursing homes from the chosen model .. 30
Figure S5 Standardized coefficients from the chosen model – number of hospital admissions by diagnosis. 31
Figure S6 Standardized coefficients from the chosen model – maximum duration of hospital stay by diagnosis. 32
Figure S7 Standardized coefficients from the chosen model – contribution of each medication to the hazard. Medication groups A through G. 33
Figure S8 Standardized coefficients from the chosen model – contribution of each medication to the hazard. Medication groups H through Y. 34
Figure S9 Average absolute and relative difference conditional on age in the life expectancy of the poorest (D1&D2) versus richest (D9&D10) who died of COVID-19 and the rest of the population in the span of 2020-2021. 39
Figure S10 Percent of the age-specific variance in life expectancy that is explained by income (defined as income quintiles). 43

S1. Description of different samples

In the main text it is mentioned that we used multiple samples for this paper's analysis, and Table S1 lists in the composition of each sample and its purpose. The cohort used for estimating survival models was comprised of 6,102,334 Dutch adults aged ≥50 as of 1 January 2012 who were followed up until death, emigration, or end of follow-up (31 December 2018). 7,028,038 Dutch adults aged ≥50 at the start of the COVID-19 pandemic were in the cohort used for generating individual-level life expectancy and years of life lost due to COVID-19 death.

Table S1 Created datasets used for analysis, validation, and results. NH = nursing home.

#	Name	Span (years)	Size (total % deaths)	% deaths Non-NH | NH	Inclusion criteria	Used for
1	Baseline	2012-2018	6,102,334 (15.7%)	13.3% | 72.9%	All people registered in the Netherlands, alive and aged 50+ as of 1-Jan-2012, who also had a recorded income in 2011.	Estimating the survival models.
2	Validation	2019	6,906,261 (6.8%)	5.6% | 56.2%	All people registered in the Netherlands, alive and aged 50+ as of 1-Jan-2019, who also had a recorded income in 2018.	Validating the survival models.
3	Prediction	2020-2021	7,028,038 (4.63%)	3.7% | 44.6%	All people registered in the Netherlands, alive and aged 50+ as of 1-Jan-2020, who also had a recorded income in 2019.	Generating and reporting results.

We ran separate survival analyses for men and women, in and out of nursing homes for each income decile. Thus, in total the Baseline sample served as the underlying dataset for 40 regressions in total. Figure S1 outlines the sample sizes for each stratified analysis.

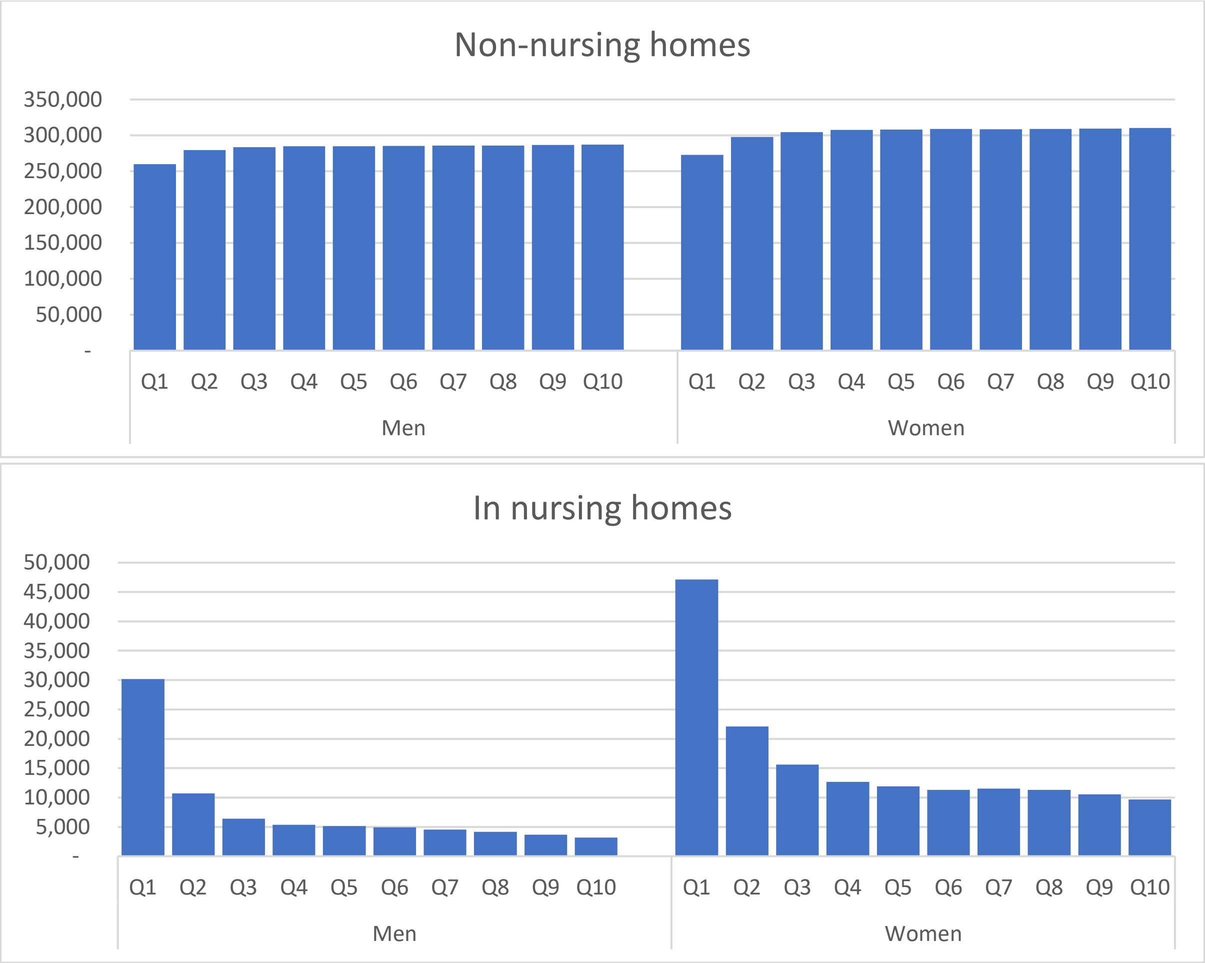

Figure S1 Baseline population size for each stratified survival analysis.

S2. Population characteristics

Table S2 Population characteristics by income deciles for the estimation and prediction samples. Income deciles were created conditional on age and sex.

Estimation sample (2012-2018), population aged 50+

	**D1 (poorest)** N = 610,383	**D2** N = 610,278	**D3** N = 610,235	**D4** N = 610,236	**D5** N = 610,166
Average age, years (Mean (SD))	64.71 (10.63)	64.71 (10.63)	64.71 (10.63)	64.71 (10.63)	64.71 (10.63)
All-cause deaths over the course of follow-up	133,674 (22%)	115,343 (19%)	104,350 (17%)	97,638 (16%)	93,355 (15%)
Lost to follow-up	12,208 (2%)	5,493 (1%)	4,272 (1%)	3,661 (1%)	3,051 (1%)
Are male	290,542 (48%)	290,492 (48%)	289,862 (48%)	289,862 (48%)	289,829 (48%)
Presence of any functional impairment	137,336 (23%)	89,101 (15%)	68,346 (11%)	60,413 (10%)	55,525 (9%)
In a nursing home	77,519 (13%)	32,955 (5%)	21,968 (4%)	18,307 (3%)	17,085 (3%)
Have used any medication	449,375 (84%)	497,774 (86%)	502,936 (86%)	503,973 (85%)	501,763 (85%)

Have been hospitalized for any reason	96,441 (16%)	103,137 (17%)	100,689 (17%)	98,858 (16%)	96,406 (16%)

	D6 N = 610,280	D7 N = 610,215	D8 N = 610,180	D9 N = 610,198	D10 (richest) N = 610,163
Average age, years (Mean (SD))	64.71 (10.63)	64.71 (10.63)	64.71 (10.63)	64.71 (10.63)	64.71 (10.63)
All-cause deaths over the course of follow-up	89,101 (15%)	86,651 (14%)	82,984 (14%)	79,326 (13%)	76,270 (13%)
Lost to follow-up	3,051 (1%)	2,441 (0.4%)	3,051 (1%)	3,051 (1%)	5,491 (1%)
Are male	290,493 (48%)	289,852 (48%)	289,836 (48%)	289,844 (48%)	289,827 (48%)
Presence of any functional impairment	51,264 (8%)	47,597 (8%)	44,543 (7%)	40,883 (7%)	37,220 (6%)
In a nursing home	16,478 (3%)	15,866 (3%)	15,255 (3%)	14,035 (2%)	12,813 (2%)
Have used any medication	499,604 (84%)	496,727 (84%)	493,035 (83%)	489,320 (82%)	483,213 (81%)
Have been hospitalized for any reason	93,983 (15%)	91,532 (15%)	89,696 (15%)	86,648 (14%)	83,592 (14%)

Prediction sample (2020-2021), population aged 50+

	D1 (poorest) N = 702,948	D2 N = 702,832	D3 N = 702,838	D4 N = 702,785	D5 N = 702,786
Average age, years (Mean (SD))	65.39 (10.7)	65.39 (10.7)	65.39 (10.7)	65.39 (10.7)	65.39 (10.7)
All-cause deaths over the course of follow-up	51,315 (7%)	42,170 (6%)	36,548 (5%)	33,031 (5%)	30,923 (4%)
Lost to follow-up	11,950 (2%)	11,245 (2%)	11,245 (2%)	11,947 (2%)	13,353 (2%)
COVID-19 deaths over the course of follow-up	7,029 (1%)	5,623 (1%)	4,217 (1%)	4,217 (1%)	3,514 (1%)
Are male	338,821 (48%)	338,765 (48%)	338,768 (48%)	338,742 (48%)	338,743 (48%)
Presence of any functional impairment	75,215 (11%)	33,736 (5%)	21,788 (3%)	17,570 (3%)	15,461 (2%)
In a nursing home	63,265 (9%)	23,193 (3%)	14,760 (2%)	11,245 (2%)	9,839 (1%)
Have used any medication	539,485 (84%)	578,323 (85%)	577,591 (84%)	576,601 (83%)	573,846 (83%)
Have been hospitalized for any reason	111,769 (16%)	116,670 (17%)	112,454 (16%)	108,229 (15%)	105,418 (15%)

	D6 N = 702,778	D7 N = 702,790	D8 N = 702,776	D9 N = 702,778	D10 (richest) N = 702,727
Average age, years (Mean (SD))	65.39 (10.7)	65.39 (10.7)	65.39 (10.7)	65.39 (10.7)	65.39 (10.7)
All-cause deaths over the course of follow-up	28,814 (4%)	28,112 (4%)	26,003 (4%)	24,597 (4%)	23,893 (3%)
Lost to follow-up	13,353 (2%)	13,353 (2%)	13,353 (2%)	12,650 (2%)	12,649 (2%)
COVID-19 deaths over the course of follow-up	3,514 (1%)	2,811 (0%)	2,811 (0%)	2,811 (0%)	2,108 (0%)
Are male	338,739 (48%)	338,745 (48%)	338,738 (48%)	338,739 (48%)	338,714 (48%)
Presence of any functional impairment	14,758 (2%)	14,759 (2%)	14,056 (2%)	12,650 (2%)	11,946 (2%)
In a nursing home	9,136 (1%)	9,136 (1%)	8,433 (1%)	7,731 (1%)	7,027 (1%)
Have used any medication	571,375 (82%)	568,068 (82%)	563,635 (81%)	558,966 (80%)	550,523 (79%)
Have been hospitalized for any reason	102,606 (15%)	99,796 (14%)	96,983 (14%)	92,767 (13%)	89,246 (13%)

In addition, the medication usage and hospitalizations by income can be found in the following tables.

Table S3 Hospitalizations in 2011 by diagnosis and income for the people in the Baseline sample. Data are clustered by income quintiles instead of income deciles for confidentiality reasons.

	Estimation sample (2012-2018), population aged 50+				
	**D1&D2 (poorest)** **N = 1,220,661**	**D3&D4** **N = 1,220,471**	**D5&D6** **N = 1,220,446**	**D7&D8** **N = 1,220,395**	**D9&d10 (richest)** **N = 1,220,361**
Infectious and parasitic diseases	2,478 (0.2%)	2,075 (0.17%)	1,892 (0.16%)	1,623 (0.13%)	1,538 (0.13%)
Neoplasms	20,690 (1.7%)	22,213 (1.82%)	21,553 (1.77%)	21,357 (1.75%)	21,491 (1.76%)
Diseases of the blood and blood-forming organs and certain disorders involving the immune mechanism	4,053 (0.33%)	3,552 (0.29%)	3,015 (0.25%)	2,758 (0.23%)	2,294 (0.19%)
Endocrine, nutritional, and metabolic diseases	4,858 (0.4%)	3,906 (0.32%)	3,503 (0.29%)	2,966 (0.24%)	2,429 (0.2%)
Mental and behavioural disorders	3,113 (0.26%)	2,478 (0.2%)	2,050 (0.17%)	1,940 (0.16%)	1,892 (0.16%)
Diseases of the nervous system	4,968 (0.41%)	5,065 (0.42%)	4,430 (0.36%)	4,125 (0.34%)	3,478 (0.29%)
Diseases of the eye and adnexa	20,495 (1.68%)	20,541 (1.68%)	20,162 (1.65%)	19,270 (1.58%)	19,001 (1.56%)
Diseases of the ear and mastoid process	1,184 (0.1%)	1,208 (0.1%)	1,147 (0.09%)	1,062 (0.09%)	964 (0.08%)
Diseases of the circulatory system	38,280 (3.14%)	38,018 (3.12%)	35,918 (2.94%)	32,816 (2.69%)	30,167 (2.47%)
Diseases of the respiratory system	12,292 (1.01%)	10,301 (0.84%)	8,702 (0.71%)	7,420 (0.61%)	6,273 (0.51%)
Diseases of the digestive system	24,377 (2%)	23,506 (1.93%)	22,127 (1.81%)	20,881 (1.71%)	19,782 (1.62%)
Diseases of the skin and subcutaneous tissue	2,869 (0.24%)	3,015 (0.25%)	2,831 (0.23%)	2,783 (0.23%)	2,843 (0.23%)
Diseases of the musculoskeletal system and connective tissue	28,808 (2.36%)	32,452 (2.66%)	31,292 (2.56%)	28,460 (2.33%)	24,786 (2.03%)
Diseases of the genitourinary system	12,915 (1.06%)	13,022 (1.07%)	12,314 (1.01%)	12,253 (1%)	11,044 (0.91%)
Congenital malformations, deformations, and chromosomal abnormalities	317 (0.03%)	293 (0.02%)	268 (0.02%)	256 (0.02%)	268 (0.02%)
Symptoms, signs, and abnormal clinical and laboratory findings, not elsewhere classified	34,838 (2.85%)	32,623 (2.67%)	30,096 (2.47%)	27,935 (2.29%)	25,176 (2.06%)
Injury, poisoning, other external causes	16,235 (1.33%)	14,755 (1.21%)	13,669 (1.12%)	13,376 (1.1%)	12,631 (1.04%)
Factors influencing health status and contact with health services	29,027 (2.38%)	30,658 (2.51%)	29,449 (2.41%)	28,069 (2.3%)	26,457 (2.17%)

Table S4 Hospitalizations in 2019 by diagnosis and income for the people in the Prediction sample who did not die from COVID-19. Data are clustered by income quintiles instead of income deciles for confidentiality reasons.

	Population unaffected by Covid-19 mortality (2020-2021), population aged 50+				
	**D1&D2 (poorest)** **N = 1,393,002**	**D3&D4** **N = 1,397,008**	**D5&D6** **N = 1,398,504**	**D7&D8** **N = 1,399,562**	**D9&D10 (richest)** **N = 1,400,699**
Infectious and parasitic diseases	4,541 (0.33%)	4,065 (0.29%)	3,440 (0.25%)	3,107 (0.22%)	2,731 (0.2%)
Neoplasms	35,522 (2.55%)	37,440 (2.68%)	36,235 (2.59%)	35,059 (2.51%)	33,827 (2.42%)
Diseases of the blood and blood-forming organs and certain disorders involving the immune mechanism	7,536 (0.54%)	6,273 (0.45%)	5,552 (0.4%)	5,024 (0.36%)	4,300 (0.31%)
Endocrine, nutritional, and metabolic diseases	6,241 (0.45%)	4,931 (0.35%)	4,209 (0.3%)	3,667 (0.26%)	2,997 (0.21%)
Mental and behavioural disorders	1,379 (0.1%)	894 (0.06%)	671 (0.05%)	602 (0.04%)	504 (0.04%)

Diseases of the nervous system	9,082 (0.65%)	8,885 (0.64%)	8,111 (0.58%)	7,586 (0.54%)	6,877 (0.49%)
Diseases of the eye and adnexa	10,712 (0.77%)	10,478 (0.75%)	10,041 (0.72%)	9,699 (0.69%)	8,768 (0.63%)
Diseases of the ear and mastoid process	1,574 (0.11%)	1,579 (0.11%)	1,482 (0.11%)	1,386 (0.1%)	1,247 (0.09%)
Diseases of the circulatory system	45,816 (3.29%)	44,048 (3.15%)	40,613 (2.9%)	37,690 (2.69%)	35,031 (2.5%)
Diseases of the respiratory system	19,209 (1.38%)	14,976 (1.07%)	12,335 (0.88%)	10,357 (0.74%)	8,712 (0.62%)
Diseases of the digestive system	39,143 (2.81%)	37,119 (2.66%)	35,494 (2.54%)	33,268 (2.38%)	31,068 (2.22%)
Diseases of the skin and subcutaneous tissue	2,619 (0.19%)	2,221 (0.16%)	2,112 (0.15%)	1,833 (0.13%)	1,709 (0.12%)
Diseases of the musculoskeletal system and connective tissue	27,122 (1.95%)	30,231 (2.16%)	28,767 (2.06%)	26,998 (1.93%)	23,616 (1.69%)
Diseases of the genitourinary system	16,451 (1.18%)	15,577 (1.12%)	14,670 (1.05%)	13,898 (0.99%)	12,578 (0.9%)
Congenital malformations, deformations, and chromosomal abnormalities	362 (0.03%)	335 (0.02%)	322 (0.02%)	322 (0.02%)	280 (0.02%)
Symptoms, signs, and abnormal clinical and laboratory findings, not elsewhere classified	26,383 (1.89%)	23,679 (1.7%)	21,523 (1.54%)	19,552 (1.4%)	17,327 (1.24%)
Injury, poisoning, other external causes	21,982 (1.58%)	20,159 (1.44%)	18,740 (1.34%)	17,956 (1.28%)	17,103 (1.22%)
Factors influencing health status and contact with health services	14,933 (1.07%)	14,990 (1.07%)	14,838 (1.06%)	13,968 (1%)	13,097 (0.94%)

Table S5 Hospitalizations in 2019 by diagnosis and income for the people in the Prediction sample who died from COVID-19. Data are clustered by income quintiles instead of income deciles for confidentiality reasons. Note: cells with NA were too small to report without risking breaking data confidentiality.

	Covid-19 decedents (2020-2021), population aged 50+				
	**D1&D2 (poorest)** **N = 12,778**	**D3&D4** **N = 8,615**	**D5&D6** **N = 7,060**	**D7&D8** **N = 6,004**	**D9&D10 (richest)** **N = 4,806**
Infectious and parasitic diseases	146 (1.14%)	96 (1.11%)	65 (0.92%)	61 (1.02%)	36 (0.75%)
Neoplasms	391 (3.06%)	361 (4.19%)	272 (3.85%)	233 (3.88%)	194 (4.04%)
Diseases of the blood and blood-forming organs and certain disorders involving the immune mechanism	197 (1.54%)	128 (1.49%)	90 (1.28%)	80 (1.33%)	56 (1.17%)
Endocrine, nutritional, and metabolic diseases	161 (1.26%)	79 (0.92%)	61 (0.86%)	43 (0.72%)	37 (0.77%)
Mental and behavioural disorders	47 (0.37%)	39 (0.45%)	26 (0.37%)	16 (0.27%)	23 (0.48%)
Diseases of the nervous system	137 (1.07%)	119 (1.38%)	91 (1.29%)	76 (1.27%)	71 (1.48%)
Diseases of the eye and adnexa	146 (1.14%)	90 (1.05%)	91 (1.29%)	77 (1.28%)	40 (0.83%)
Diseases of the ear and mastoid process	15 (0.12%)	13 (0.15%)	14 (0.2%)	NA	NA
Diseases of the circulatory system	840 (6.57%)	642 (7.45%)	570 (8.07%)	433 (7.21%)	335 (6.97%)
Diseases of the respiratory system	631 (4.94%)	498 (5.78%)	348 (4.93%)	237 (3.95%)	178 (3.7%)
Diseases of the digestive system	377 (2.95%)	291 (3.38%)	228 (3.23%)	200 (3.33%)	151 (3.14%)
Diseases of the skin and subcutaneous tissue	43 (0.34%)	30 (0.35%)	35 (0.5%)	18 (0.3%)	17 (0.35%)
Diseases of the musculoskeletal system and connective tissue	225 (1.76%)	205 (2.38%)	156 (2.21%)	112 (1.87%)	77 (1.6%)
Diseases of the genitourinary system	348 (2.72%)	254 (2.95%)	188 (2.66%)	164 (2.73%)	126 (2.62%)
Congenital malformations, deformations, and chromosomal abnormalities	NA	NA	NA	NA	NA
Symptoms, signs, and abnormal clinical and laboratory findings, not elsewhere classified	422 (3.3%)	305 (3.54%)	229 (3.24%)	184 (3.07%)	140 (2.91%)
Injury, poisoning, other external causes	489 (3.83%)	386 (4.48%)	318 (4.5%)	270 (4.5%)	233 (4.85%)
Factors influencing health status and contact with health services	191 (1.5%)	146 (1.7%)	119 (1.69%)	104 (1.73%)	80 (1.67%)

Table S6 Medication usage by income across different samples – Women. Note: cells with NA were too small to report without risking breaking data confidentiality.

Estimation sample (2012-2018), women aged 50+

ATC 1st level	D1&D2 (poorest) N = 571,001	D3&D4 N = 611,924	D5&D6 N = 616,894	D7&D8 N = 617,335	D9&D10 (richest) N = 619,861
A	307,313 (54%)	307,553 (50%)	291,791 (47%)	273,047 (44%)	252,221 (41%)
B	144,292 (25%)	150,411 (25%)	140,467 (23%)	127,974 (21%)	117,216 (19%)
C	301,660 (53%)	322,362 (53%)	309,866 (50%)	288,851 (47%)	261,767 (42%)
D	161,365 (28%)	166,505 (27%)	162,613 (26%)	157,729 (26%)	155,337 (25%)
G	48,649 (9%)	50,239 (8%)	50,277 (8%)	50,498 (8%)	54,114 (9%)
H	99,525 (17%)	104,517 (17%)	97,037 (16%)	88,588 (14%)	79,280 (13%)
J	193,170 (34%)	203,220 (33%)	199,072 (32%)	194,584 (32%)	194,946 (31%)
L	19,757 (3%)	22,519 (4%)	22,455 (4%)	22,162 (4%)	22,067 (4%)
M	185,119 (32%)	188,167 (31%)	182,539 (30%)	174,829 (28%)	164,201 (26%)
N	214,468 (38%)	200,160 (33%)	182,662 (30%)	164,396 (27%)	145,233 (23%)
P	9,536 (2%)	9,056 (1%)	9,007 (1%)	8,951 (1%)	9,546 (2%)
R	182,092 (32%)	182,537 (30%)	173,286 (28%)	164,767 (27%)	156,825 (25%)
S	125,392 (22%)	127,831 (21%)	128,437 (21%)	127,974 (21%)	129,923 (21%)
V	1,713 (0.3%)	1,591 (0.26%)	1,481 (0.24%)	1,420 (0.23%)	1,364 (0.22%)
Y	8,394 (1%)	7,160 (1%)	6,354 (1%)	5,741 (1%)	5,269 (1%)

Women unaffected by COVID-19 mortality (2020-2021), aged 50+

ATC 1st level	D1&D2 (poorest) N = 673,318	D3&D4 N = 707,969	D5&D6 N = 713,236	D7&D8 N = 714,010	D9&D10 (richest) N = 716,403
A	375,442 (56%)	356,038 (50%)	332,511 (47%)	309,024 (43%)	280,257 (39%)
B	183,883 (27%)	183,010 (26%)	169,679 (24%)	155,654 (22%)	140,272 (20%)
C	345,479 (51%)	351,365 (50%)	333,367 (47%)	311,665 (44%)	279,469 (39%)
D	176,948 (26%)	174,160 (25%)	169,750 (24%)	166,364 (23%)	163,268 (23%)
G	47,671 (7%)	46,726 (7%)	45,932 (6%)	45,839 (6%)	49,647 (7%)
H	139,713 (21%)	138,479 (20%)	129,310 (18%)	118,169 (17%)	105,526 (15%)
J	208,123 (31%)	211,754 (30%)	206,482 (29%)	200,851 (28%)	199,733 (28%)
L	25,451 (4%)	28,673 (4%)	29,243 (4%)	28,846 (4%)	28,799 (4%)
M	172,302 (26%)	166,302 (23%)	159,908 (22%)	152,941 (21%)	142,421 (20%)
N	242,529 (36%)	218,054 (31%)	196,354 (28%)	176,860 (25%)	155,889 (22%)
P	12,254 (2%)	10,903 (2%)	10,485 (1%)	10,210 (1%)	10,030 (1%)
R	225,494 (33%)	217,913 (31%)	205,983 (29%)	196,139 (27%)	186,910 (26%)
S	152,305 (23%)	146,691 (21%)	145,857 (20%)	145,872 (20%)	148,081 (21%)
V	1,953 (0.29%)	1,558 (0.22%)	1,355 (0.19%)	1,214 (0.17%)	1,146 (0.16%)
Y	16,496 (2%)	12,956 (2%)	11,126 (2%)	9,711 (1%)	8,597 (1%)

Women unaffected by COVID-19 mortality (2020-2021), aged 50+

ATC 1st level	D1&D2 (poorest) N = 2,701	D3&D4 N = 2,436	D5&D6 N = 2,048	D7&D8 N = 1,643	D9&D10 (richest) N = 1,331
A	2,335 (86%)	2,144 (88%)	1,796 (88%)	1,391 (85%)	1,099 (83%)
B	1,708 (63%)	1,567 (64%)	1,331 (65%)	1,027 (63%)	793 (60%)
C	2,183 (81%)	2,048 (84%)	1,692 (83%)	1,327 (81%)	1,008 (76%)
D	1,076 (40%)	965 (40%)	812 (40%)	627 (38%)	0,530 (40%)

G	259 (10%)	218 (9%)	192 (9%)	157 (10%)	129 (10%)
H	1,008 (37%)	973 (40%)	750 (37%)	572 (35%)	440 (33%)
J	1,396 (52%)	1,315 (54%)	1,073 (52%)	850 (52%)	693 (52%)
L	190 (7%)	188 (8%)	149 (7%)	116 (7%)	95 (7%)
M	853 (32%)	768 (32%)	656 (32%)	497 (30%)	369 (28%)
N	1,479 (55%)	1,295 (53%)	1,063 (52%)	818 (50%)	609 (46%)
P	60 (2%)	48 (2%)	40 (2%)	31 (2%)	21 (2%)
R	1,213 (45%)	1,067 (44%)	846 (41%)	634 (39%)	455 (34%)
S	903 (33%)	813 (33%)	721 (35%)	555 (34%)	437 (33%)
V	49 (2%)	28 (1%)	15 (1%)	17 (1%)	NA
Y	271 (10%)	255 (10%)	204 (10%)	156 (9%)	138 (10%)

Table S7 Medication usage by income across different samples – Men.

Estimation sample (2012-2018), men aged 50+

ATC 1st level	D1&D2 (poorest)<br><br>N = 539,529	D3&D4<br><br>N = 568,518	D5&D6<br><br>N = 570,265	D7&D8<br><br>N = 571,571	D9&D10 (richest)<br><br>N = 573,441
A	249,370 (46%)	239,119 (42%)	221,434 (39%)	205,423 (36%)	189,981 (33%)
B	168,441 (31%)	173,341 (30%)	162,468 (28%)	151,695 (27%)	141,812 (25%)
C	272,138 (50%)	287,045 (50%)	277,434 (49%)	266,581 (47%)	251,225 (44%)
D	129,163 (24%)	130,361 (23%)	126,770 (22%)	125,574 (22%)	126,558 (22%)
G	52,982 (10%)	56,170 (10%)	56,342 (10%)	56,414 (10%)	56,541 (10%)
H	55,032 (10%)	55,089 (10%)	48,986 (9%)	44,525 (8%)	39,166 (7%)
J	141,896 (26%)	144,972 (26%)	139,601 (24%)	137,234 (24%)	140,493 (25%)
L	14,405 (3%)	16,430 (3%)	16,138 (3%)	16,175 (3%)	16,400 (3%)
M	141,465 (26%)	145,768 (26%)	137,092 (24%)	128,489 (22%)	118,645 (21%)
N	144,917 (27%)	121,947 (21%)	105,556 (19%)	92,652 (16%)	79,766 (14%)
P	4,154 (1%)	4,093 (1%)	4,049 (1%)	4,172 (1%)	5,046 (1%)
R	138,767 (26%)	138,434 (24%)	130,420 (23%)	125,460 (22%)	121,225 (21%)
S	89,238 (17%)	89,883 (16%)	88,334 (15%)	88,479 (15%)	88,539 (15%)
V	2,320 (0.43%)	1,990 (0.35%)	1,768 (0.31%)	1,715 (0.3%)	1,663 (0.29%)
Y	7,176 (1%)	5,799 (1%)	4,733 (1%)	4,287 (1%)	3,613 (1%)

Men unaffected by COVID-19 mortality (2020-2021), aged 50+

ATC 1st level	D1&D2 (poorest)<br>N = 639,042	D3&D4<br>N = 665,607	D5&D6<br>N = 668,091	D7&D8<br>N = 669,512	D9&D10 (richest)<br>N = 671,468
A	311,852 (49%)	286,144 (43%)	262,693 (39%)	244,907 (37%)	221,920 (33%)
B	210,053 (33%)	208,734 (31%)	195,216 (29%)	184,049 (27%)	171,426 (26%)
C	329,362 (52%)	335,532 (50%)	322,488 (48%)	309,515 (46%)	288,664 (43%)
D	143,209 (22%)	137,914 (21%)	134,487 (20%)	134,237 (20%)	135,704 (20%)
G	69,208 (11%)	68,358 (10%)	67,410 (10%)	67,286 (10%)	67,080 (10%)
H	78,410 (12%)	76,478 (11%)	68,947 (10%)	62,934 (9%)	55,463 (8%)
J	150,367 (24%)	148,430 (22%)	142,237 (21%)	139,727 (21%)	141,545 (21%)
L	18,213 (3%)	20,900 (3%)	21,379 (3%)	21,357 (3%)	22,024 (3%)
M	138,289 (22%)	139,711 (21%)	131,681 (20%)	123,592 (18%)	114,351 (17%)
N	175,481 (27%)	136,915 (21%)	117,250 (18%)	104,645 (16%)	90,245 (13%)

P	5,496 (1%)	4,992 (1%)	4,743 (1%)	4,687 (1%)	4,969 (1%)
R	166,151 (26%)	158,548 (24%)	151,456 (23%)	146,556 (22%)	143,023 (21%)
S	106,081 (17%)	103,236 (16%)	101,951 (15%)	101,565 (15%)	103,473 (15%)
V	2,684 (0.42%)	1,997 (0.3%)	1,804 (0.27%)	1,607 (0.24%)	1,544 (0.23%)
Y	13,228 (2%)	9,052 (1%)	7,349 (1%)	6,561 (1%)	5,506 (1%)

Men unaffected by COVID-19 mortality (2020-2021), aged 50+

ATC 1st level	D1&D2 (poorest) N = 4,477	D3&D4 N = 3,784	D5&D6 N = 3,092	D7&D8 N = 2,579	D9&D10 (richest) N = 2,013
A	3,597 (80%)	3,073 (81%)	2,406 (78%)	1,970 (76%)	1,510 (75%)
B	3,038 (68%)	2,682 (71%)	2,143 (69%)	1,745 (68%)	1345 (67%)
C	3,609 (81%)	3,131 (83%)	2,472 (80%)	2,032 (79%)	1,520 (76%)
D	1,717 (38%)	1472 (39%)	1101 (36%)	909 (35%)	733 (36%)
G	1127 (25%)	1010 (27%)	826 (27%)	686 (27%)	521 (26%)
H	1,190 (27%)	1066 (28%)	792 (26%)	638 (25%)	471 (23%)
J	1,960 (44%)	1,671 (44%)	1,310 (42%)	1080 (42%)	851 (42%)
L	311 (7%)	305 (8%)	258 (8%)	216 (8%)	171 (8%)
M	1181 (26%)	1019 (27%)	751 (24%)	611 (24%)	518 (26%)
N	1,833 (41%)	1,457 (39%)	1,110 (36%)	871 (34%)	657 (33%)
P	60 (1%)	38 (1%)	34 (1%)	28 (1%)	16 (1%)
R	1,799 (40%)	1,526 (40%)	1161 (38%)	908 (35%)	682 (34%)
S	1302 (29%)	1044 (28%)	907 (29%)	729 (28%)	583 (29%)
V	102 (2%)	60 (2%)	50 (2%)	32 (1%)	39 (2%)
Y	269 (6%)	263 (7%)	181 (6%)	171 (7%)	146 (7%)

S3. Detailed methodology of life expectancy prediction

Study design

We estimated individual-level life years lost due to COVID-19 in several steps. First, we estimated Cox proportional hazards models based on administrative data linked to death records. We used different automated variable selection techniques and sets of covariates, where the outcome of interest was all-cause mortality over a 7-year follow-up (2012-2018). We followed (Breslow, 1972) in order get a non-parametric estimate of the baseline hazard functions across the observed follow-up time. For each model, we assumed a log-linear relationship between the baseline hazard function and time for its extrapolation beyond the observed follow-up time, but we also accounted for seasonality. Next, we predicted the all-cause hazard for all people aged 50+ who were alive on 1-Jan-2020 and combined it with the estimate of the baseline survival curve over the next 50 years. The outcome was an individual-level survival curve, which once integrated over time resulted in an individual-level remaining life expectancy for each person of the Dutch population. Out of the whole population, we identified those who died of COVID-19 in the span of 2020-2021 and took their counterfactual LE estimate to be the years of life lost due to the virus. This resulted in a distribution of LE for the entire population, and more specifically, YLL when looking at COVID-19 decedents. Then, we compared the YLL distributions' characteristics and dispersion to the general population. Lastly, we investigated the ability of income to explain the heterogeneity in YLL.

Model estimation

The first step is to estimate the hazard ratio for each individual using different types of models and combinations of variables. For ease of notation, going forward when we mention type of model, we mean the type of regression or regularization method used, and analysis refers to the variables that we include in the models. We obtained the annual disposable household income from the tax registry (corrected for the size of the household using an equivalence scale estimated and used by Statistics Netherlands (Siermann et al., 2004)), and defined income deciles conditional on 1-year age groups and sex. We ran each model/analysis for men/women in and out of nursing homes and for each income decile separately. Thus, each row in Table S8 was run 40 times using the Baseline sample as documented in Table S1 and Figure S1. We grouped hospitalizations

into 18 groups, based on the International shortlist for hospital morbidity tabulation (ISHMT) (*Eurostat/OECD/WHO IHMT*, n.d.), hence when we mention “by diagnosis” we mean by either one of the 18 diagnoses groups. We discarded hospitalizations that occurred for conditions originating in the perinatal period (ISHMT Chapter 16) and related to pregnancy (Chapter 15).

While it is likely that machine learning methods such as random survival forests (RSF) can outperform regularization methods in terms of prediction (Deryugina et al., 2019), they come with certain limitations. Namely, we aimed to utilize the entirety of our population-wide administrative dataset for estimation (as opposed to sampling) which is a more difficult computational endeavour using RSF. Our main reason, however, was that our study necessitated extrapolation for a long period of time - 50 years in total. As such, extrapolating the baseline survival for an additional 43 years based on a 7-year period (as is the case for LASSO/Elastic Net) would yield greater accuracy if the entire population is considered, rather than relying on the survival curve slope of each individual separately (RSF).

Table S8 Types of regression and analyses.

		**non-Nursing homes**		**Nursing homes**	
**Analysis**	**Model**	**Main variables**	**N variables**	**Main variables**	**N variables**
Basic 1	Regular Cox PH	1-year age groups (categorical), all medications (ATC2), type of functional impairment, hospitalizations by diagnosis and urgency	179	5-year age groups, type of nursing home care, type of functional impairment, hospitalizations by diagnosis and urgency	61
Basic 1	Cox-LASSO/ Cox-Elastic Net				
Basic 2	Cox-LASSO/ Cox-Elastic Net	1-year age groups (categorical), all medications (ATC2), type of functional impairment, hospitalizations by diagnosis and urgency, and number of admissions by diagnosis	197	5-year age groups, type of nursing home care, type of functional impairment, hospitalizations by diagnosis and urgency, and number of admissions by diagnosis	79
Basic 3	Cox-LASSO/ Cox-Elastic Net	1-year age groups (categorical), all medications (ATC2), type of functional impairment, hospitalizations by diagnosis and urgency, number of admissions by diagnosis, duration of hospital stay by diagnosis.	215	5-year age groups, type of nursing home care, type of functional impairment, hospitalizations by diagnosis and urgency, and number of admissions by diagnosis, duration of hospital stay by diagnosis.	97

- Number of admissions - this gives us how many times was someone admitted for the same ISHMT group within the calendar year.

- Urgency - this gives us the maximum urgency by diagnosis. If someone was admitted multiple times within the year for the same ISHMT group and all were non-urgent, then the urgency is 1. If someone was admitted multiple times within the year for the same ISHMT group and at least one was urgent, then the urgency for that ISHMT equals 2. 92% (2011) and 81% (2019) of the urgent admissions were also the last admission for that person for that ISHMT in the year.
- Duration of hospital stay - this gives the number of days by ISHMT group a person was hospitalized for. If multiple hospitalizations occurred by ISHMT group, then the variable takes the value of the longest stay. For 89% (2011) and 87% (2019) of the people their longest stay was also their last admission for that ISHMT group in the year.

Regular Cox model

The Cox proportional hazards model assumed that the hazard rate of death of each individual can be represented by some baseline hazard that is either lowered of increased by the influence of the covariates:

$$h(t_i|x_i,\beta) \;=\; h_0(t_i) * exp(x_i\beta)$$

Where the individual's hazard rate, $h(t_i|x_i,\beta)$, at time t_i, is dependent on the baseline hazard rate (h_0) at the same time, but also on the vector of covariates the individual possesses. The vector of coefficients β is the set of betas that minimize the negative partial log-likelihood function:

$$\ln L\,(\beta) = \sum_{i=1}^{N} \delta_i \left[x_i\beta \;-\; \ln \sum_{j\in R(t_i)} exp\big(x_j\beta\big) \right] \qquad (C1)$$

Where δ_i is in an indicator variable equal to one for individuals where we observe an event (all-cause death), and zero otherwise. The observations in the risk set $R(t_k) \;=\; \{i{:}\,T *\geq t_k\}$ are all observations whose true event time is after t_k and are hence still alive at t_k. So, observations whose deaths we do not observe during the follow-up contribute to the likelihood function only by being part of the alive risk set, indexed by j in (C1).

We ran the regular Cox model in STATA using the *stcox* command.

Regularization models

The LASSO and Elastic Net are dimensionality reduction techniques that minimize the same function but with a penalty. The function that they minimize can be summarized as:

$$\ln L\,(\beta) = \sum_{i=1}^{N} \delta_i \left[x_i\beta \; - \; \ln \sum_{j \in R(t_i)} exp\big(x_j\beta\big) \right] + \lambda\{(1-\alpha) * \|\beta\|_2^2 \; + \; \alpha * \|\beta\|_1\} \qquad (C2)$$

Where we have two parameters that need to be chosen - the penalty term (λ) and α.

The latter is user-selected and by choosing its value, we perform L1 regularization when alpha equals one (LASSO), or L2 regularization (Ridge regression) when alpha is zero. When $\alpha = 0.5$, the algorithm uses both L1 and L2 features, which overcomes their individual limitations, and is called Elastic Net. In particular, because LASSO adds a penalty term to the likelihood function proportional to the sum of the absolute value of the coefficients, it has the property to set some of the coefficients to exactly zero. This results in sparse models where only a subset of the variables has non-zero coefficients, making it useful for variable selection. However, when there are correlated predictors, LASSO can struggle, as it tends to arbitrarily pick only one of them and ignore the others. The L2 regularization, also known as Ridge, adds a penalty term proportional to the square of the sum of the coefficients, and shrinks them towards zero. This helps to avoid overfitting and stabilize the model, but it doesn't perform variable selection, as it never sets any coefficients to exactly zero. Hence, Ridge will keep all of the variables that are given in the input and calculate coefficients for them, even if their effects are so small that they are virtually irrelevant. Finally, Elastic Net combines the L1 and L2 regularization techniques and can balance the trade-off between sparsity and stability and handle correlated predictors (James et al., 2021). We chose two distinct values for the alpha - $\alpha = 1$ (LASSO) and $\alpha = 0.5$ (Elastic Net) and ran both variations to check which fits our data better.

The lambda (λ), also called tuning parameter, is responsible for the size of the coefficients, and by extension – which variables (features) get chosen by the models. A different set of coefficient estimates will be produced for each value of lambda. Hence, selecting a good value for it is crucial, and cross-validation provides a solution to this

problem. In short, in cross-validation, we take a grid of λ values, then the sample is randomly split into k-folds (in practice, 5- up to 10-folds). The model gets estimated on the k-1 folds and validated on the last fold. This process gets repeated k times until all folds have been used for validation at least once. Then, we pick whichever tuning parameter produces the smallest cross-validation error. We used *glmnet* package in R with command *cv.glmnet* in order to perform 5-k fold cross-validation. The command directly estimates the model as well, so it can be readily applied for prediction.

Model performance and external validation

After the models were estimated, we generated model performance measures on the same training datasets (apparent validation). This is to assess how each one is able to correctly predict the risk of death of individual stemming from the same dataset used for estimating the model. We used the validation sample, as defined in Table S1, to generate metrics about how well each one performed on brand new data.

Discrimination

Discrimination speaks to how well a model is able to separate predictions between those with and without the event. We used Harrell's C-index as a measure of discrimination, which is calculable by the *somersd* command in STATA (Newson, 2010). Harrell's C-index can be biased by the distribution of censoring (Uno et al., 2011), but in our study, this is less of a concern because we observe the entire population from the same starting point and the censoring is likely non-informative. Unlike studies with significant loss to follow-up over time, we maintain a sufficient number of participants and events over seven years, minimizing the impact of censoring on our results.

- Harrell C-Index – also known as the concordance index, taking censoring into account. The advantage here is that both regular Cox in STATA and Cox-LASSO/Elastic Net in R produce this statistic directly. For each observation i, the model predicts a certain risk of event, let's call it PI_i for prognostic index, which is just the sum of the covariates weighted by the beta coefficients produced by the model, $PI_i = \sum_{j=1}^{k} \beta_j * x_j$. If the model is any good then observations with a shorter time-to-event T*, should also have a higher prognostic index. The C-index itself measures how often does this hold true for two randomly paired

observations plus half the probability that the hazards in the randomly two paired observations are equal. The higher C-index, the better.

$$C = Pr(PI(i,T*) > PI(j,T) \mid T* < T) + \frac{Pr(PI(i,T*) = PI(j,T) \mid T* < T)}{2}$$

We used the exact same samples to generate regular Cox and machine learning model results. Thus, in order to be consistent, we predicted the response and link functions of each LASSO and Elastic Net model from R and imputed the results into STATA. Then we applied the *somersd* command. Table S9 displays the discrimination statistics results from each model. For ease of display, we averaged out the performance measures across income deciles, hence we present the metrics only for men and women, in- and out of nursing homes.

Table S9 Ability or each model to discriminate observations with/without the event. Numbers in bold are the best across all models for the group.

**Validation dataset**		**C-index**				
		**Non-nursinghome**		**Nursinghome**		**Average**
**Model**	**Analysis**	**Women**	**Men**	**Women**	**Men**	
Regular Cox	Basic 1	0.8832	0.8652	0.6156	0.6441	0.7520
LASSO	Basic 1	0.8837	0.8653	0.6145	0.6438	0.7518
Elastic Net	Basic 1	0.8837	0.8653	0.6149	0.6453	0.7523
LASSO	Basic 2	0.8850	0.8657	0.6156	0.6461	0.7531
Elastic Net	Basic 2	0.8851	**0.8659**	0.6156	0.6455	0.7530
LASSO	Basic 3	0.8852	0.8657	0.6157	0.6480	0.7536
Elastic Net	Basic 3	**0.8853**	0.8658	**0.6165**	**0.6482**	**0.7539**

It appears that numbers are not that different across models/analyses, so we can say that all models appear to have equally good discrimination and neither model/analysis is immediately superior to another. However, we chose to proceed with the Elastic Net (α=0.5), analysis Basic 3, as it encompasses the full set of hospitalization variables as well as has a high discrimination capability across groups.

Calibration

Calibration tells us how well the predicted risks agree with the observed events. For example, those who did die should have a higher hazard compared to alive. In other

words, this is the accuracy of prediction and when it comes to survival – it is the survival probabilities at any time after t=0.

Calibration slope - the calibration slope as per (Royston, 2014) is a method to see how well the individual event probabilities estimated at different time points match reality. This can be done in STATA using the *stcoxcal* command and it can be run both on training and validation datasets across all types of models. It can evaluate both regular and LASSO/Elastic Net regressions since its required input is the PI_i only and both models use the exact same training dataset[1]. The algorithm works in several steps:

1. For the training dataset, demean the log of the estimated hazard ratio ($\ln HR_i$) and denote the newly de-meaned variable as $\widetilde{xb}$.
   For the training dataset, estimate the following regression.
   $$h(t_i|x_i, \beta = 1) = h_0(t_i) * exp(1 * \widetilde{xb})$$
   Get the log baseline cumulative hazard function from this model, $\ln H_0$.
2. Model $\ln H_0$ as a second-degree fractional polynomial in t and extrapolate the log baseline cumulative hazard function on the out-of-sample failure times, i.e., on the validation dataset.
3. On the validation dataset, predict event probabilities from the model $\hat{F}_i(t)$ at the selected times t={1, ... k} by:
   $$\hat{F}_i(t) = 1 - \hat{S}_0(t)^{HR_i} \equiv ln(-ln(1 - \hat{F}_i(t))) = xb_i + ln\, H_0$$
   Convert $\hat{F}_i(t)$ to the log cumulative hazard function by:
   $$ln\hat{H}(t, x_i) = ln(-ln(1 - \hat{F}_i(t)))$$
4. Obtain pseudo-values the cumulative failure probabilities, $\tilde{F}_i(t)$.
5. The coefficients to be tested come from the equation of the form:

$$\tilde{F}_i(t) = \gamma_0 + \gamma_1 * ln\hat{H}(t, x_i) \qquad (C3)$$

Ultimately, we test:

- Test 1 - Intercept test. We wish to know if $\gamma_0 = 0$, constraining $\gamma_1 = 1$. If the test is significant, it means that $\gamma_0 \neq 0$ and so we have a calibration error known as

[1] Note: In order for the performance measures to be comparable between regular Cox and Cox-ML, they must use the same training dataset (which they do) because functions in STATA that can calculate Harrell's C, calibration slope and Royston's R2 1) use estimates of the predicted hazard ratio 2) estimate the baseline hazard function and extrapolate it into new datapoints.

"miscalibration in the large". Such an error speaks to lack of agreement of the predicted and observed survival fraction in the external validation data set. It indicates systematic underprediction or overprediction, stemming from overall difference in the true baseline survival between training and validation datasets. In our setting, we estimate:

$$\tilde{F}_i(t) = \Upsilon_{01} * I(t=1) + \Upsilon_{02} * I(t=4) + 1 * lnH_i(t)$$
$$Test\ 1: \Upsilon_{01} = \Upsilon_{02} = 0$$

- Test 2 – Slope test. Test of $\gamma_1 = 1$ with γ_0 already estimated. If the test is insignificant, then $\gamma_1 = 1$ and we have good calibration; When the test is significant, then $\gamma_1 = 0$ which implies a complete lack of model discrimination. The case when $\gamma_1 < 1$ also indicates reduced discrimination in the validation dataset, likely due to overfitting in the training dataset with the spread of predictions being too wide: the predictions are too low for low-risk observations and too high for high-risk observations.

$$\tilde{F}_i(t) = \Upsilon_{01} * I(t=1) + \Upsilon_{02} * I(t=4) + \Upsilon_1 * lnH_i(t)$$
$$Test\ 2: \Upsilon_1 = 1$$

- Test 3 – Joint test. This examines the overall evidence for (linear) miscalibration. If we are concerned about type 1 error, a conservative approach is to perform the joint test first, then proceed to the separate tests only if the result of the joint test is significant. Ideally, this test is insignificant.

$$\tilde{F}_i(t) = \Upsilon_{01} * I(t=1) + \Upsilon_{02} * I(t=4) + \Upsilon_1 * lnH_i(t)$$
$$Test\ 3: \left| \begin{matrix} \Upsilon_{01} = \Upsilon_{02} = 0 \\ \Upsilon_1 = 1 \end{matrix} \right.$$

- Test 4 – trend test. This is used when we simultaneously wish to test survival at multiple timepoints. Tests whether the slope over all timepoints equals 1. If the test is insignificant, then we can conclude that the model is well calibrated in the validation dataset over selected all timepoints. We can only test within the model timeframe, in our case the maximum t=4 (for 2016-2019 analysis), but we are specifically more interested in t=1 and t=4 (this will be t=8 for the 2012-2019 analysis).

$$\tilde{F}_i(t) = \Upsilon_0 * t + \Upsilon_1 * lnH_i(t) + \Upsilon_2 * lnH_i(t) * t$$
$$Test\ 4: \Upsilon_2 = 0$$

More detailed explanation is available in (Royston, 2014). In summary, $\tilde{F}_i(t)$ are the pseudo-observations (observed cumulative failure probabilities corrected for censoring). They are jackknife quantities and do not bear a resemblance to identifiable event probabilities when considered individually (Parner & Andersen, 2010). Due to the leave-one-out way of estimating the pseudo values, they are not constrained to the interval [0,1], however, their usefulness lies in their unbiasedness in the expected values. The model is well calibrated for any value t if the following holds:

$$E\{\tilde{F}_i(t)\} = F\{t; x_i\}$$

with F{.} being the observed cumulative failure probability at t for an observation with covariate set x_i

Under that condition, the equation (C3) fits a generalized linear model (GLM) well. For example, let's say we have the vector j={T_1, T_2, T_3, ... T_J} with district failure times from the dataset. We also have the vector t={1, ... k} with the user-selected times at which we want to check calibration. Also, the pseudo-cumulative failure probabilities, $\tilde{F}_i(t)$, are the inverse of the pseudo-corrected Kaplan-Meier (KM) survival curve for observation i, which is given by:

$$\tilde{S}_{KM}[t = k; i(T = j)] = n_j * S_{KM}(t = k) - (n_j - 1) * S_{KM}(t = k; -i)$$

Where $S_{KM}(t = k)$ is the total Kaplan-Meier curve at time k. If k is not part of the unique failure times of the original dataset, then the survival value is taken as the one closest to but still lower than k. $S_{KM}(t = k; -i)$ is the same at the total KM curve but without the ith observation. The "weight" here is the n_j, which denotes how many observations at unique failure time j are either right censored or have experienced the event.

Table S10 displays the calibration slopes on the validation dataset with slopes being tested hallway throughout the year (day 183) and on the last day of the year. Additionally, we tested whether the intercept is equal to zero (Test 1), and if the slope is equal to one (Test 2), however these tests are not displayed only discussed in the following section.

Table S10 Calibration slope averaged out across income deciles for ease of presentation.

Validation dataset		Slope				
		**Non-nursinghome**		**Nursinghome**		**Average**
**Model**	**Analysis**	**Women**	**Men**	**Women**	**Men**	

Regular Cox	Basic 1	0.9614	**1.0056**	0.7395	0.9511	0.9144
LASSO	Basic 1	0.9638	1.0084	0.7579	1.0531	0.9458
Elastic Net	Basic 1	0.9635	1.0080	0.7595	**1.0367**	0.9419
LASSO	Basic 2	0.9639	1.0077	0.7702	1.1901	0.9830
Elastic Net	Basic 2	0.9648	1.0076	0.7775	1.1800	0.9825
LASSO	Basic 3	0.9643	1.0045	0.7830	1.1688	0.9802
Elastic Net	Basic 3	**0.9651**	1.0063	**0.7901**	1.1987	**0.9901**

Judging from Table S10 we observe an acceptable agreement between observed and model-predicted events halfway throughout the year 2019 and at its end. Elastic Net with all variables seems to provide the best calibration for people outside of nursing homes, although regardless of method/analysis people in Q6 and Q7 had an intercept that was statistically different from zero. However, its slope was statistically equal to one across all stratified groups. For people in nursing homes all models yielded a slope equal to zero across the same number of groups, but its intercept was statistically equal to one across the most groups.

The trend test was insignificant across all models and analysis regardless of sex and nursing home usage, with the exception of men not nursing homes from Q5. This suggests that there is no interaction between the selected times and the calibration slope.

Baseline hazard function and extrapolation

Deryugina et al., 2019 estimate the baseline hazard function, $h_0(t)$ for the observed timeframe, by following (Breslow, 1972):

$$\widehat{h_0}(t_i) = \frac{d_{t_i}}{\sum_{j\in R(t_i)} HR_j}$$

$$\widehat{H}_0(t_K) = \sum_{k=0}^{K} \widehat{h_0}(t_k)$$

Where d_{t_i} are the deaths that occur at timepoint t_i, and the denominator is the sum of the model-estimated hazard ratios for the people who are alive at timepoint t_i.

Depending on what interval is chosen, one can obtain estimates for the baseline hazard function on a monthly, quarterly, yearly, or any other desired interval. Since a smaller time interval yields more accurate estimates of $h_0(t_i)$, we chose to go with a 30-day interval. We assumed a log-linear relationship for extrapolation beyond t_{max}, but also corrected for seasonality:

$$lnh_0(t_i) = \beta_0 + \beta_1 * t + \beta_2 * Month$$

This linearity assumption seems to hold when plotting the actual baseline hazards $lnh_0(t_i)$ versus the estimated $\widehat{lnh_0(t_i)}$ across time (see Figure S2).

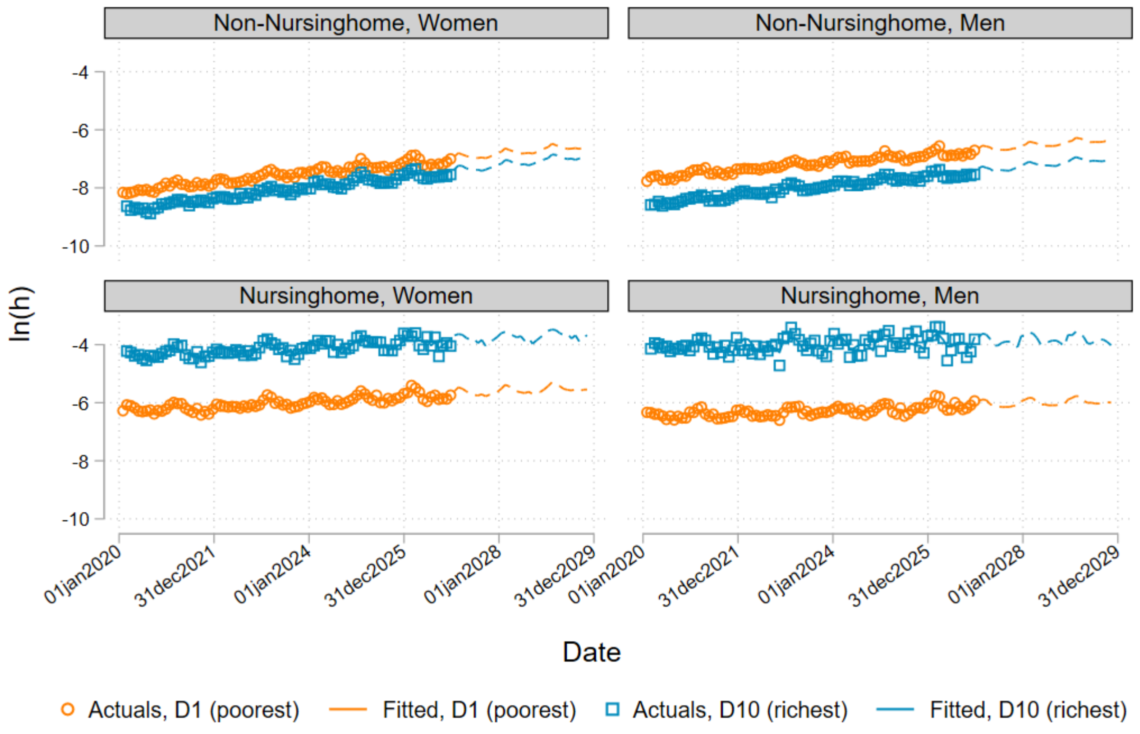

Figure S2 Natural logarithm of the baseline hazard function across time. D1 and D10 here represent the poorest and richest 10% of the population.

Elastic Net coefficients

Before the Elastic Net penalty is applied and the likelihood function minimized to produce the best beta coefficients, the algorithm standardizes the underlying dataset in the background (default setting standardize = TRUE in the *glmnet* package) and solves the equation. However, before reporting the coefficients they get returned back to the

original scale. Hence, in order to get an idea about proper variable importance, we normalized them. In the following figures the absolute size of each coefficient can be interpreted as variable importance. The more away from zero a coefficient, the greater its contribution to the overall change in the hazard. Also, positive coefficients effect survival negatively and vice-versa. Tables S11-S12 display more succinctly which variables were selected by the Elastic Net, and the following figures show the standardized coefficients.

Table S11 Variables that were selected (denoted by X) by the Elastic Net for the final analysis; For the poorest (D1&D2) and richest (D9&D10) two income deciles separately for men and women who were not in a nursing home.

	Male				Female			
**Coefficient**	**D1**	**D2**	**D9**	**D10**	**D1**	**D2**	**D9**	**D10**
Age*	X	X	X	X	X	X	X	X
Presence of funtional impaiment								
Somatic	X	X	X	X	X	X		X
Psychogeriatric								
Psychiatric	X	X	X	X	X		X	X
Physical	X	X	X		X	X	X	
Other								
Hospitalizations								
Infectious and parasitic diseases								
Number of admissions	X			X	X			
Duration of admission	X		X	X	X		X	X
High urgency of admission	X	X	X		X			X
Neoplasms								
Number of admissions	X	X	X	X	X	X	X	X
Duration of admission	X	X	X	X	X		X	X
High urgency of admission	X	X	X	X	X	X	X	X
Diseases of the blood and blood-forming organs and certain disorders involving the immune mechanism								
Number of admissions	X	X	X		X	X	X	X
Duration of admission		X	X	X	X		X	X
High urgency of admission		X	X	X	X	X		X
Endocrine, nutritional, and metabolic diseases								
Number of admissions	X	X	X	X	X		X	X
Duration of admission	X	X	X	X	X		X	X
High urgency of admission	X	X	X	X	X	X		X
Mental and behavioural disorders								
Number of admissions	X		X	X	X		X	
Duration of admission		X		X			X	X
High urgency of admission	X	X	X	X	X	X	X	X
Diseases of the nervous system								
Number of admissions	X				X		X	X
Duration of admission	X		X	X				X
High urgency of admission	X	X	X	X	X		X	X

Diseases of the eye and adnexa								
Number of admissions	X		X	X	X		X	
Duration of admission			X	X	X			
High urgency of admission	X	X			X			X
Diseases of the ear and mastoid process								
Number of admissions	X		X					
Duration of admission	X	X	X		X		X	
High urgency of admission				X	X		X	X
Diseases of the circulatory system								
Number of admissions	X	X	X	X				
Duration of admission	X	X	X	X	X	X	X	
High urgency of admission	X		X	X			X	
Diseases of the respiratory system								
Number of admissions	X	X	X	X	X	X	X	X
Duration of admission	X	X	X	X	X	X	X	X
High urgency of admission	X	X	X	X	X	X		X
Diseases of the digestive system								
Number of admissions	X			X	X		X	X
Duration of admission		X	X				X	X
High urgency of admission	X			X	X		X	X
Diseases of the skin and subcutaneous tissue								
Number of admissions			X					X
Duration of admission	X		X	X	X	X	X	X
High urgency of admission	X	X	X	X	X	X	X	
Diseases of the musculoskeletal system and connective tissue								
Number of admissions			X		X			X
Duration of admission	X	X	X	X	X	X	X	X
High urgency of admission	X		X	X	X		X	X
Diseases of the genitourinary system								
Number of admissions			X				X	X
Duration of admission	X	X	X	X	X		X	X
High urgency of admission	X	X	X	X	X	X	X	X
Congenital malformations, deformations, and chromosomal abnormalities								
Number of admissions							X	
Duration of admission	X		X	X	X		X	X
High urgency of admission		X		X	X			
Symptoms, signs, and abnormal clinical and laboratory findings, not elsewhere classified								
Number of admissions	X		X	X	X		X	X
Duration of admission	X	X	X	X	X		X	X
High urgency of admission		X		X	X		X	X
Injury, poisoning, other external causes								
Number of admissions	X	X	X	X	X		X	
Duration of admission	X	X	X	X			X	X
High urgency of admission	X							X
Factors influencing health status and contact with health services								
Number of admissions	X	X	X	X	X	X	X	X
Duration of admission	X	X	X	X	X	X	X	X
High urgency of admission			X	X	X		X	X

Medications (ATC 2nd level)								
A01				X	X		X	X
A02	X	X		X	X	X	X	X
A03	X	X	X	X	X	X	X	X
A04	X	X	X	X	X	X	X	X
A05	X	X	X	X	X	X	X	X
A06	X	X	X	X	X	X		X
A07	X	X	X	X	X	X	X	X
A08				X				
A09	X	X	X	X	X	X	X	X
A10	X	X	X	X	X	X	X	X
A11	X	X	X	X	X	X	X	X
A12	X	X	X	X	X	X	X	
A14	X	X	X	X	X			X
A16	X		X	X				X
B01	X	X	X	X	X	X	X	X
B02	X	X	X	X	X	X	X	X
B03	X	X	X	X	X	X	X	X
B05	X	X	X	X	X	X	X	X
B06					X			
C01	X	X	X	X	X	X	X	X
C02	X	X	X	X	X	X	X	X
C03	X	X	X	X	X	X	X	X
C04	X	X	X	X	X		X	X
C05	X	X	X	X	X			X
C07	X	X	X	X	X	X	X	X
C08		X	X	X	X	X	X	X
C09	X	X	X	X	X	X	X	X
C10	X	X	X	X	X	X	X	X
D01	X	X	X	X	X		X	X
D02	X	X	X	X	X	X	X	X
D03	X	X	X	X	X		X	X
D04	X	X	X		X			X
D05	X		X	X			X	X
D06	X	X	X	X		X	X	X
D07	X	X	X	X	X	X	X	X
D08	X	X	X		X		X	X
D09	X		X	X	X	X	X	X
D10	X	X	X	X	X		X	
D11	X	X			X	X	X	X
G01	X	X	X		X			
G02			X	X	X			
G03		X	X	X	X	X	X	X
G04	X	X	X	X		X	X	X
H01	X	X	X	X	X		X	X
H02	X	X	X	X	X	X	X	X
H03		X	X	X	X	X	X	
H04	X			X	X	X	X	

H05	X	X	X		X	X	X	X
J01	X	X	X	X	X	X	X	X
J02	X	X	X	X	X		X	X
J04	X	X	X	X			X	X
J05	X	X		X			X	X
J06	X		X	X	X		X	X
J07	X			X			X	X
L01	X	X	X	X	X	X	X	X
L02	X	X	X	X	X	X	X	X
L03	X	X	X	X	X	X	X	X
L04	X	X	X	X	X	X	X	X
M01	X	X	X	X	X	X	X	X
M02	X		X		X		X	X
M03	X	X	X	X	X	X	X	X
M04	X	X	X	X	X	X	X	X
M05	X	X	X	X	X	X	X	X
M09					X			
N01	X	X	X	X	X	X	X	X
N02	X	X	X	X	X	X	X	X
N03	X	X	X	X	X	X	X	X
N04	X	X	X	X	X	X	X	X
N05	X	X	X	X	X	X	X	X
N06	X	X	X	X	X	X	X	X
N07	X		X	X	X		X	X
P01	X	X	X	X	X			X
P02	X	X	X	X			X	
P03	X		X	X				
R01	X	X	X	X	X	X	X	X
R02	X			X	X		X	
R03	X	X	X	X	X	X	X	X
R05	X	X	X		X	X	X	X
R06	X	X	X	X	X	X	X	X
S01	X	X	X	X	X	X	X	X
S02	X	X	X	X	X	X	X	X
V01	X	X	X		X		X	
V03	X	X	X	X	X	X	X	X
V04				X				X
V06		X			X			X
V07	X	X	X	X	X	X	X	X
Y	X	X	X	X	X	X	X	X

* In the analysis age is a 1-year categorical variable, and all ages except one were selected, serving as a reference group

Table S12 Variables that were selected (denoted by X) by the Elastic Net for the final analysis; For the poorest (D1&D2) and richest (D9&D10) two income deciles separately for men and women who were in a nursing home.

	Male				Female			
**Coefficient**	**D1**	**D2**	**D9**	**D10**	**D1**	**D2**	**D9**	**D10**
Age*	X	X	X	X	X	X	X	X
Presence of funtional impaiment								
Somatic								X
Psychogeriatric			X					
Psychiatric						X		
Physical			X					X
Other					X	X		
Hospitalizations								
Infectious and parasitic diseases								
Number of admissions	X				X	X		
Duration of admission				X		X		X
High urgency of admission								
Neoplasms								
Number of admissions	X	X	X		X	X	X	
Duration of admission		X	X			X		
High urgency of admission	X	X	X	X	X	X	X	X
Diseases of the blood and blood-forming organs and certain disorders involving the immune mechanism								
Number of admissions	X	X			X	X	X	
Duration of admission		X						
High urgency of admission	X					X	X	
Endocrine, nutritional, and metabolic diseases								
Number of admissions	X				X	X		
Duration of admission	X	X						
High urgency of admission	X	X			X	X		
Mental and behavioural disorders								
Number of admissions		X		X		X		
Duration of admission				X		X		
High urgency of admission	X							
Diseases of the nervous system								
Number of admissions				X				
Duration of admission	X							
High urgency of admission					X	X	X	
Diseases of the eye and adnexa								
Number of admissions		X				X		X
Duration of admission								
High urgency of admission		X						
Diseases of the ear and mastoid process								
Number of admissions	X			X				
Duration of admission		X		X				
High urgency of admission				X				
Diseases of the circulatory system								
Number of admissions	X		X	X	X			X
Duration of admission	X		X	X	X			
High urgency of admission	X			X	X			
Diseases of the respiratory system								

Number of admissions	X		X	X	X	X	X	X
Duration of admission		X						
High urgency of admission	X	X		X	X	X		
Diseases of the digestive system								
Number of admissions	X							
Duration of admission	X				X	X		
High urgency of admission	X	X				X		
Diseases of the skin and subcutaneous tissue								
Number of admissions				X	X		X	
Duration of admission				X	X			
High urgency of admission	X							
Diseases of the musculoskeletal system and connective tissue								
Number of admissions		X	X		X			X
Duration of admission	X	X			X	X	X	X
High urgency of admission	X			X	X		X	X
Diseases of the genitourinary system								
Number of admissions								
Duration of admission	X	X					X	
High urgency of admission	X	X			X	X		
Congenital malformations, deformations, and chromosomal abnormalities								
Number of admissions		X						
Duration of admission		X						
High urgency of admission					X			
Symptoms, signs, and abnormal clinical and laboratory findings, not elsewhere classified								
Number of admissions				X	X	X	X	
Duration of admission		X		X				X
High urgency of admission	X		X		X	X		
Injury, poisoning, other external causes								
Number of admissions						X	X	X
Duration of admission	X	X		X	X	X		X
High urgency of admission	X	X	X	X	X		X	X
Factors influencing health status and contact with health services								
Number of admissions	X	X			X		X	X
Duration of admission					X	X		X
High urgency of admission		X		X				
Nursing home admission package								
Intensity: Low level care	X	X	X	X	X	X	X	X
Intensity: High level care	X	X	X	X	X	X	X	X
Intensity: Very high level care	X	X	X	X	X	X	X	X
Intensity: Special (GGZ)	X	X		X	X	X		
Type: Nursing and care (VV)	X	X		X	X	X		
Type: Mental disability (VG)	X	X		X	X	X		
Type: Slight mental disability (LVG)						X		
Type: Physical disability (LG)								
Type: Sensory impairment (ZG)	X	X	X	X	X	X	X	X
Type: Mental health (GGZ)	X	X		X	X	X		

* In the analysis age is a 5-year categorical variable, and all ages except one were selected, serving as a reference group

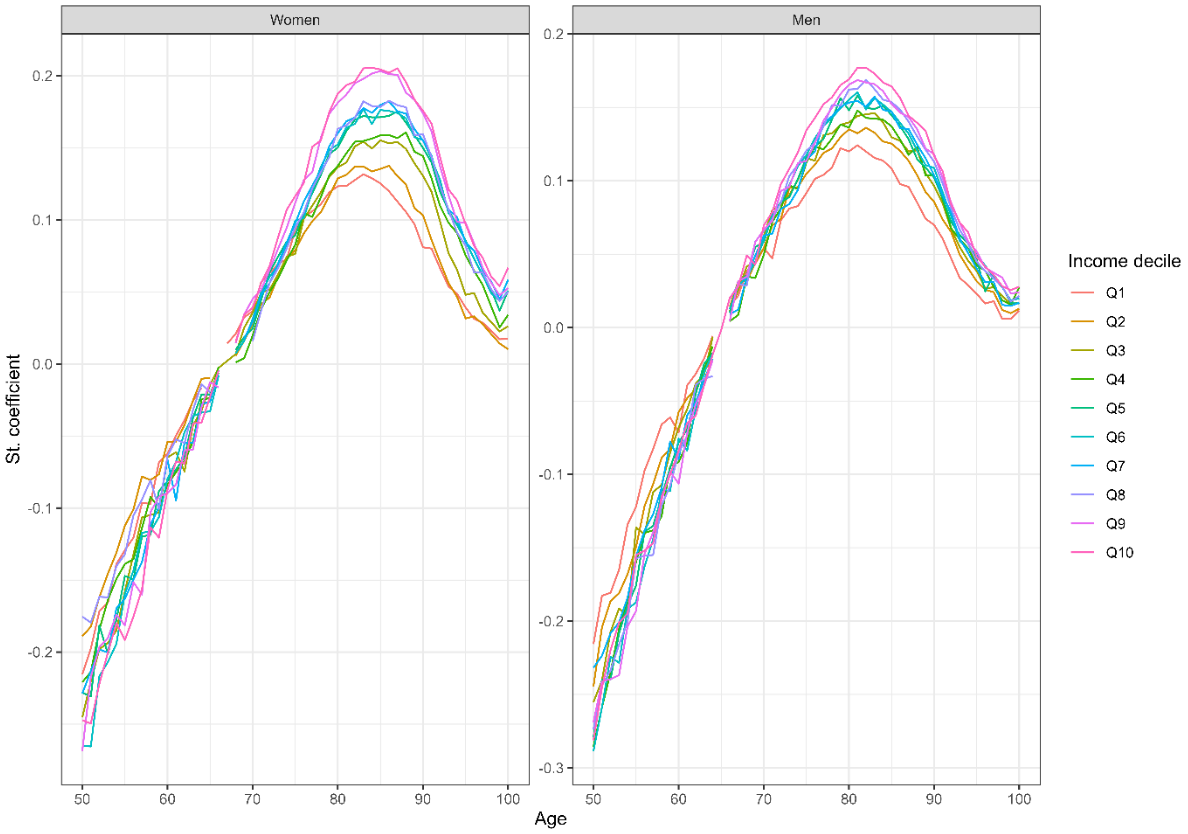

Figure S3 Standardized age coefficients for people not in nursing homes from the chosen model. Q1-Q10 represent income deciles.

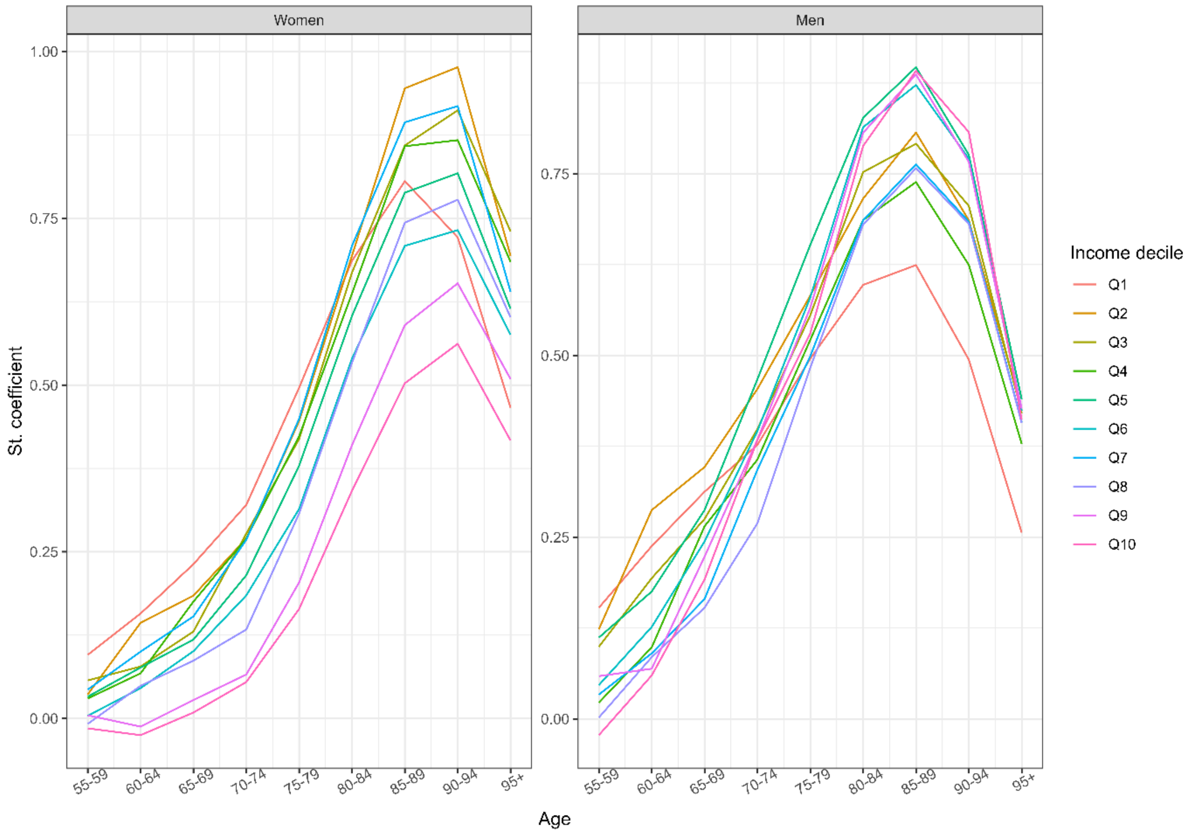

Figure S4 Standardized age coefficients for people in nursing homes from the chosen model. Q1-Q10 represent income deciles.

The following labels are applicable to the hospitalization graphs.

DIAG1	Infectious and parasitic diseases
DIAG2	Neoplasms
DIAG3<br>DIAG4	Diseases of the blood and blood-forming organs and certain disorders involving the immune mechanism<br>Endocrine, nutritional, and metabolic diseases
DIAG5	Mental and behavioural disorders
DIAG6	Diseases of the nervous system
DIAG7	Diseases of the eye and adnexa
DIAG8	Diseases of the ear and mastoid process
DIAG9	Diseases of the circulatory system
DIAG10	Diseases of the respiratory system
DIAG11	Diseases of the digestive system
DIAG12	Diseases of the skin and subcutaneous tissue
DIAG13	Diseases of the musculoskeletal system and connective tissue
DIAG14	Diseases of the genitourinary system
DIAG15	Congenital malformations, deformations, and chromosomal abnormalities
DIAG16	Symptoms, signs, and abnormal clinical and laboratory findings, not elsewhere classified
DIAG17	Injury, poisoning, other external causes
DIAG18	Factors influencing health status and contact with health services

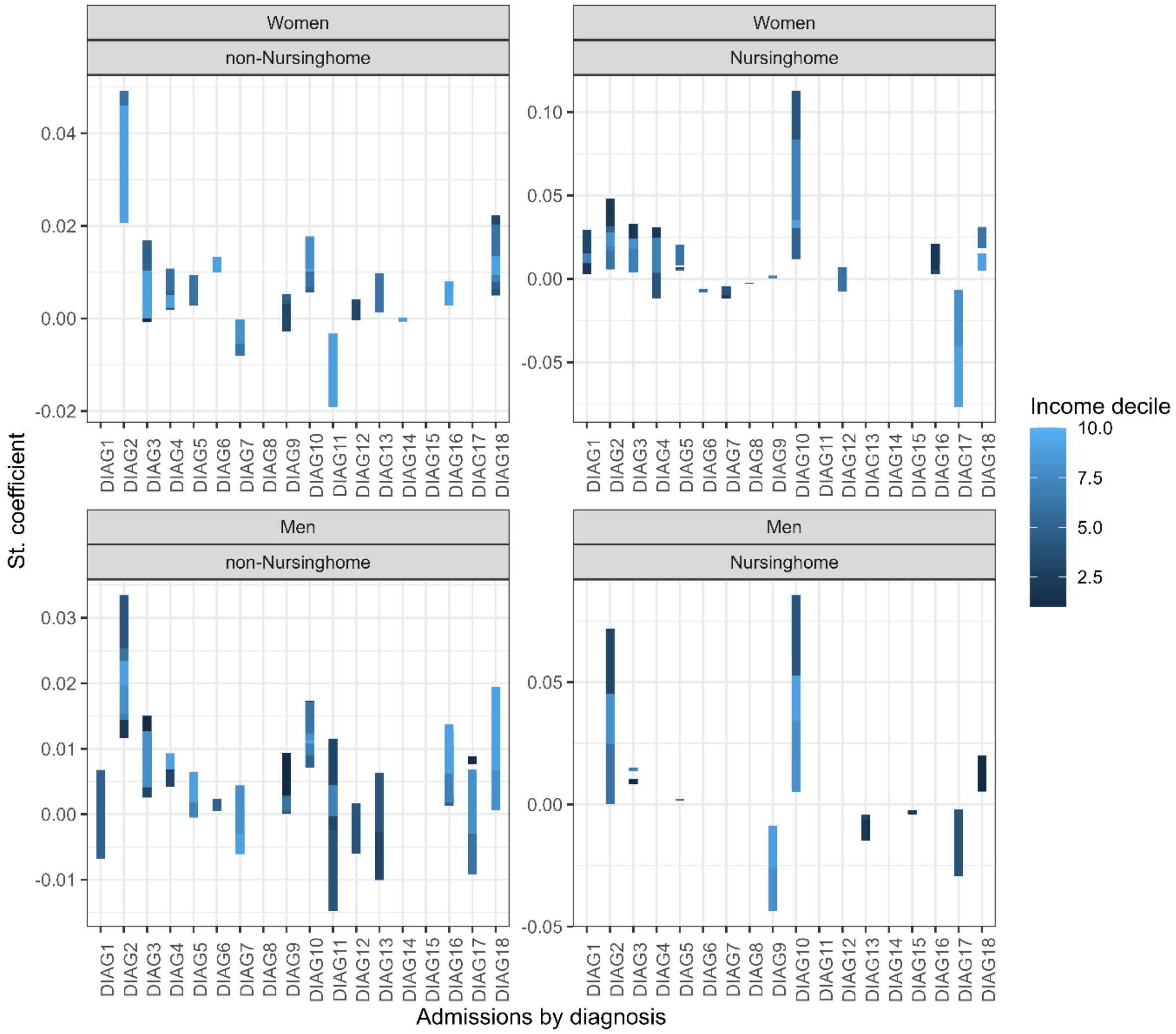

Figure S5 Standardized coefficients from the chosen model – number of hospital admissions by diagnosis.

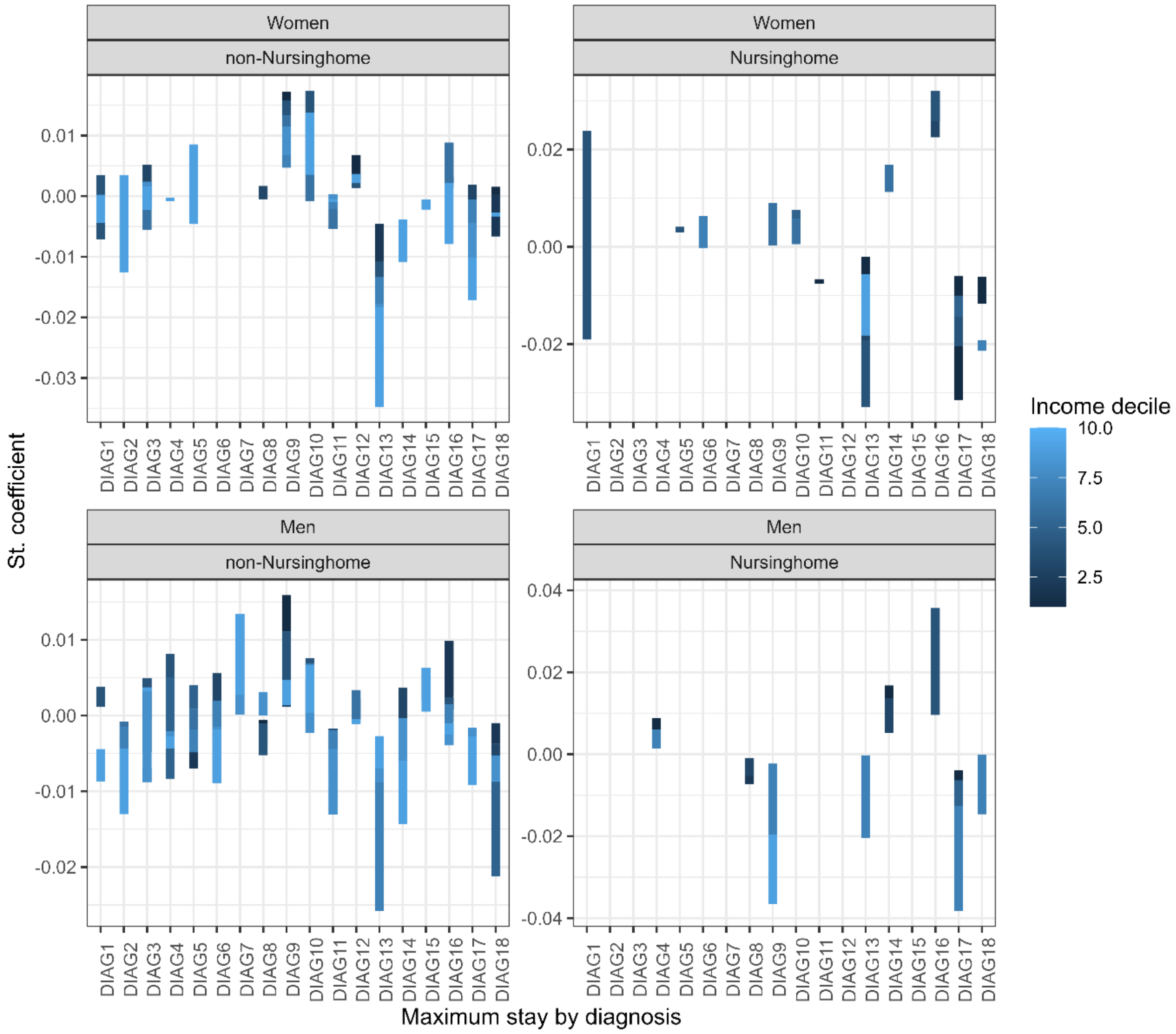

Figure S6 Standardized coefficients from the chosen model – maximum duration of hospital stay by diagnosis.

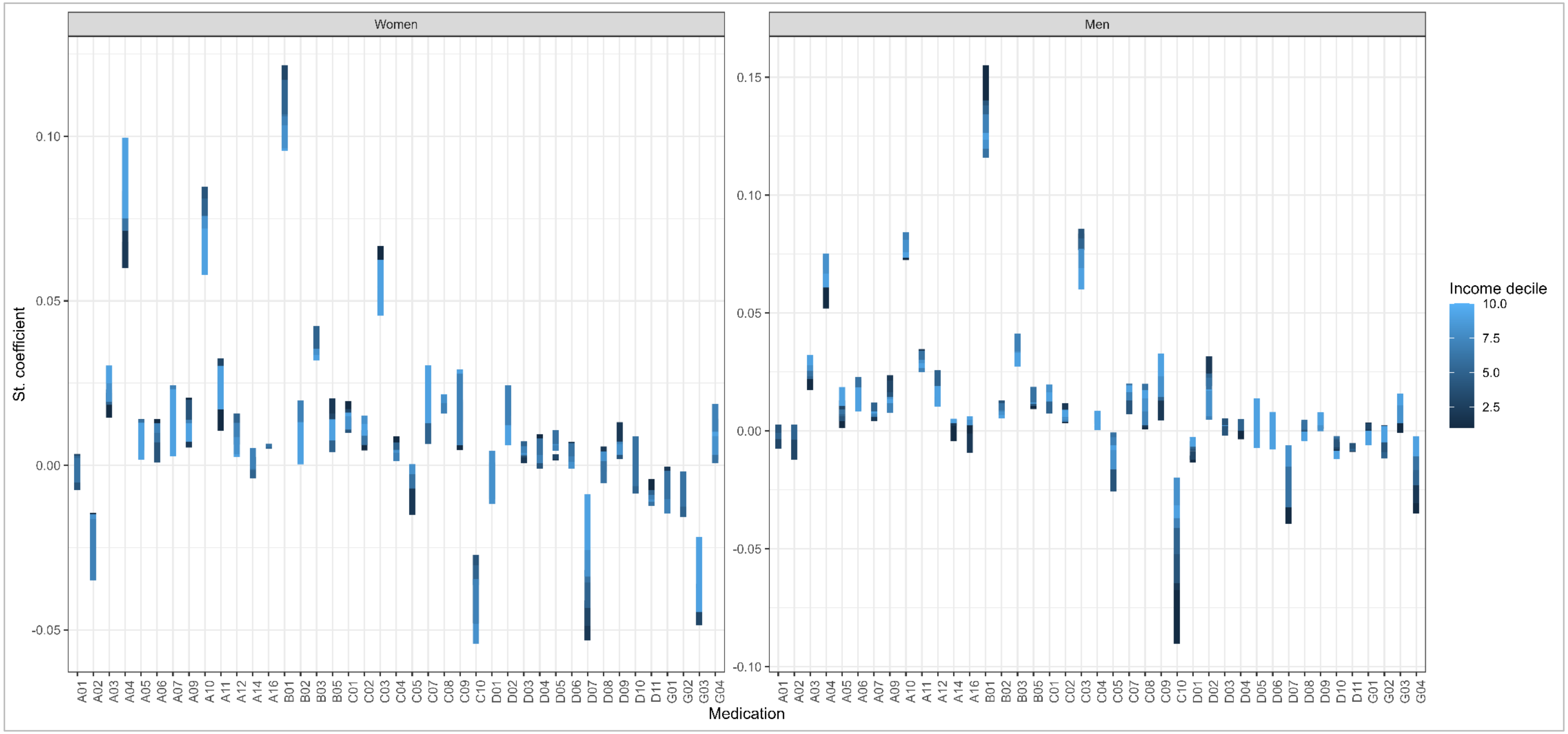

Figure S7 Standardized coefficients from the chosen model – contribution of each medication to the hazard. Medication groups A through G.

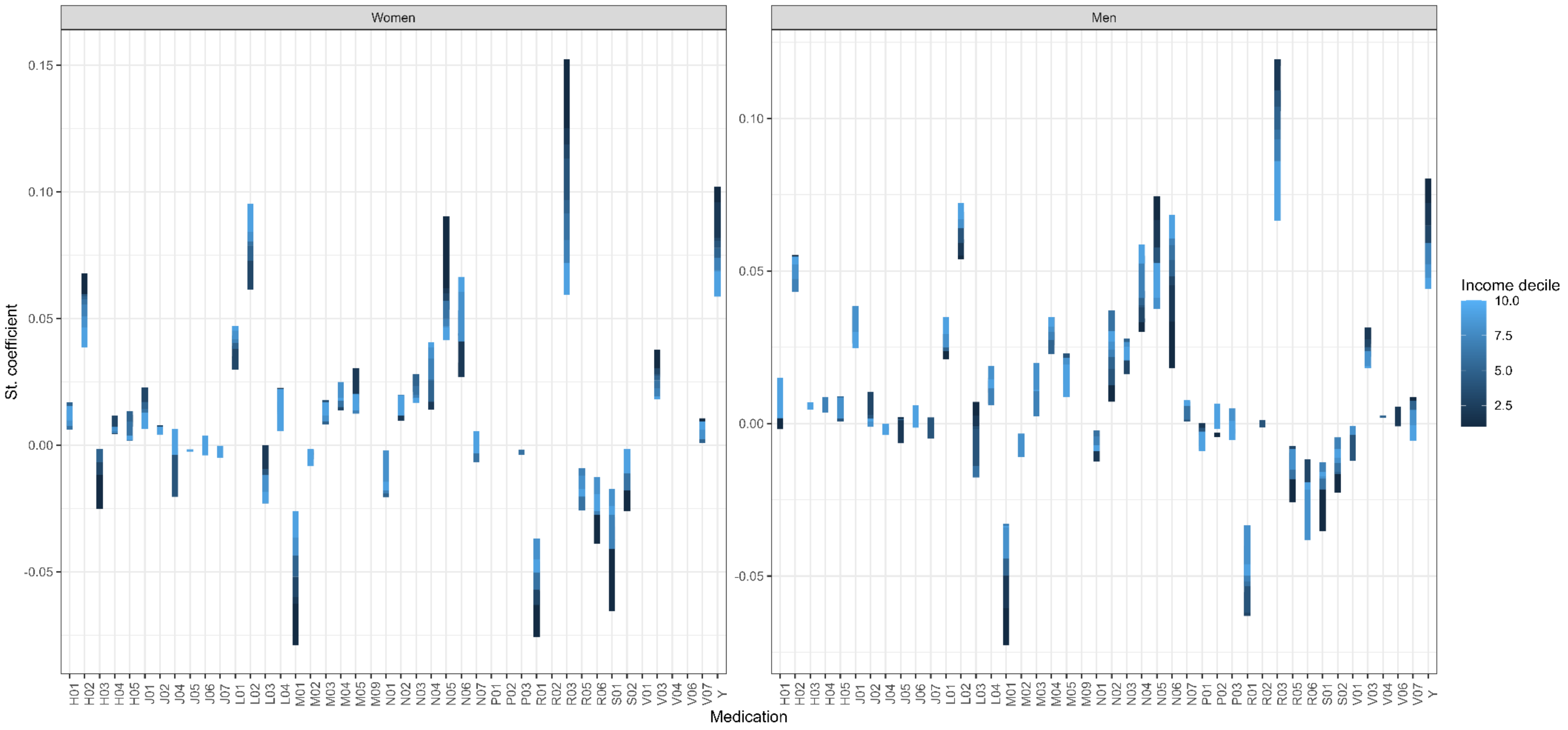

Figure S8 Standardized coefficients from the chosen model – contribution of each medication to the hazard. Medication groups H through Y.

S4. More life expectancy results

The absolute difference in life expectancy (LE) across income is largest at younger ages: average LE at 60 is 17.2 (SD 2.6) for the lowest incomes and 21.1 (SD 1.7) for the highest, and those numbers at 85 are 6 (SD 2.5) versus 7 (SD 2). The relative difference varies – the lowest income group's average LE is 18.4% at 60, 15.2% at 75, 13.4% at 85, and 18.2% at 90 lower than that of the highest.

In contrast, among COVID-19 decedents the LE distributions are very similar between low and high incomes. Low-income individuals had a much higher probability of dying from COVID-19 which is reflected in the greater frequency of the LE distributions. However, the shapes of the distributions are very similar. Moreover, the two income groups lost the same number of YLL per COVID-19 death across all ages combined and their variation was similar - 6.38 (SD 4.4), CVQ 0.52 for D1&D2 and 6.3 (SD 4.7), CVQ 0.5 for D9&D10. At age 60 these numbers were 13.5 (SD 4.4) versus 16.5 (SD 4.3) and at age 85 – 4 (SD 2) versus 4.7 (SD 2.3), for the poorest and richest income groups, respectively.

Table S13 Predicted life expectancy distribution characteristics across all income groups, 2020-2021.

Group	Age	Mean	SD	Median	IQR	CV	CVQ	Skewness	Kurtosis	Size (N)
COVID-19 decedents	All ages	6.54	4.56	5.20	6.07	0.70	0.51	1.20	4.07	39,263
	Age 60	14.93	4.63	16.24	6.37	0.31	0.21	-0.69	2.53	217
	Age 75	8.24	3.47	8.65	6.15	0.42	0.38	-0.08	1.87	1,115
	Age 85	4.56	2.20	4.06	3.50	0.48	0.38	0.54	2.18	1,777
	Age 90	3.27	1.63	2.37	2.24	0.50	0.34	0.87	2.76	1,384
Everyone else	All ages	16.68	5.73	17.50	8.19	0.34	0.24	-0.52	2.60	6,988,775
	Age 60	19.20	2.43	19.65	2.45	0.13	0.06	-1.72	8.61	234,534
	Age 75	11.94	2.59	12.47	3.16	0.22	0.13	-1.22	4.71	146,765
	Age 85	6.69	2.19	7.09	3.10	0.33	0.23	-0.52	2.45	63,290
	Age 90	4.60	1.88	4.80	3.39	0.41	0.38	-0.06	1.86	29,737
Other-cause death	All ages	7.49	5.13	6.12	7.14	0.68	0.52	1.00	3.40	285,953
	Age 60	14.80	5.03	16.05	7.35	0.34	0.24	-0.64	2.46	2,892
	Age 75	8.61	3.71	9.02	6.37	0.43	0.37	-0.18	1.96	7,778
	Age 85	4.93	2.29	4.72	3.87	0.47	0.40	0.32	2.02	10,662

	Age 90	3.56	1.73	3.02	2.69	0.49	0.38	0.65	2.35	9,067
Population	All ages	16.62	5.77	17.46	8.27	0.35	0.24	-0.52	2.59	7,028,038
	Age 60	19.19	2.43	19.65	2.45	0.13	0.06	-1.72	8.64	234,751
	Age 75	11.92	2.62	12.45	3.19	0.22	0.13	-1.22	4.67	147,880
	Age 85	6.64	2.22	7.03	3.17	0.33	0.23	-0.50	2.38	65,067
	Age 90	4.54	1.89	4.73	3.48	0.42	0.40	-0.02	1.84	31,121

Table S14 Predicted life expectancy distribution characteristics across by income. D1&D21 = first two deciles (poorest), D9&D10 – last two deciles (richest), 2020-2021.

Income	Metric	Age	Mean	SD	Median	IQR	CV	CVQ	Skewness	Kurtosis	Size (N)
D1&D2 (poorest)	COVID-19 decedents	All ages	6.38	4.44	4.94	6.12	0.70	0.52	1.11	3.70	12,778
		Age 60	13.45	4.40	13.29	6.24	0.33	0.23	-0.36	2.36	103
		Age 75	7.48	3.39	7.41	5.81	0.45	0.41	0.20	1.98	381
		Age 85	4.04	2.09	2.91	3.17	0.52	0.40	0.92	2.73	528
		Age 90	2.95	1.54	2.26	1.56	0.52	0.27	1.39	4.04	382
	Everyone else	All ages	15.10	5.32	15.87	7.56	0.35	0.25	-0.53	2.60	1,393,002
		Age 60	17.22	2.62	18.13	2.57	0.15	0.07	-1.62	6.69	46,848
		Age 75	10.93	2.83	11.48	3.53	0.26	0.16	-0.89	3.60	29,203
		Age 85	6.13	2.47	6.43	4.14	0.40	0.34	-0.14	1.94	12,489
		Age 90	4.01	1.99	3.63	3.43	0.50	0.44	0.46	1.97	5,843
	Other-cause death	All ages	7.62	5.16	6.33	7.91	0.68	0.56	0.81	2.78	80,516
		Age 60	13.63	4.36	14.40	6.77	0.32	0.24	-0.58	2.43	1,139
		Age 75	7.84	3.49	7.97	6.01	0.44	0.39	-0.01	1.94	2,174
		Age 85	4.40	2.27	3.55	3.19	0.52	0.36	0.73	2.53	2,670
		Age 90	3.09	1.61	2.26	1.82	0.52	0.29	1.23	3.61	2,183
	Population	All ages	15.02	5.38	15.82	7.64	0.36	0.25	-0.53	2.58	1,405,780
		Age 60	17.21	2.63	18.13	2.58	0.15	0.07	-1.63	6.69	46,951
		Age 75	10.88	2.87	11.44	3.57	0.26	0.16	-0.89	3.54	29,584
		Age 85	6.04	2.49	6.33	4.32	0.41	0.37	-0.11	1.90	13,017
		Age 90	3.94	1.98	3.48	3.37	0.50	0.43	0.51	2.02	6,225
D3&D4	COVID-19 decedents	All ages	6.76	4.53	5.55	6.28	0.67	0.51	1.10	3.81	8,615
		Age 60	15.44	5.09	17.87	5.95	0.33	0.18	-1.06	2.72	50
		Age 75	8.03	3.28	8.13	5.56	0.41	0.36	-0.01	1.93	281
		Age 85	4.70	2.18	4.44	3.37	0.46	0.36	0.49	2.41	394
		Age 90	3.28	1.55	2.60	2.15	0.47	0.32	0.73	2.50	282
	Everyone else	All ages	16.17	5.54	17.04	8.06	0.34	0.24	-0.53	2.57	1,397,008

		Age 60	18.58	2.07	19.04	1.94	0.11	0.05	-2.38	12.89	46,900
		Age 75	11.47	2.59	11.93	3.17	0.23	0.14	-1.04	4.34	29,290
		Age 85	6.63	2.16	6.95	3.07	0.33	0.23	-0.46	2.46	12,618
		Age 90	4.55	1.85	4.70	3.28	0.41	0.37	0.00	1.93	5,942
	Other-cause death	All ages	7.67	5.11	6.41	7.20	0.67	0.50	0.93	3.24	61,003
		Age 60	14.60	5.09	16.16	7.61	0.35	0.25	-0.84	2.62	612
		Age 75	8.52	3.66	8.86	5.95	0.43	0.35	-0.21	2.08	1,792
		Age 85	5.03	2.25	4.88	3.80	0.45	0.39	0.26	2.05	2,191
		Age 90	3.61	1.73	3.23	2.63	0.48	0.37	0.61	2.38	1,838
	Population	All ages	16.11	5.59	17.00	8.13	0.35	0.25	-0.53	2.56	1,405,623
		Age 60	18.58	2.08	19.04	1.94	0.11	0.05	-2.39	12.96	46,950
		Age 75	11.44	2.62	11.90	3.19	0.23	0.14	-1.04	4.29	29,571
		Age 85	6.57	2.19	6.90	3.12	0.33	0.23	-0.43	2.41	13,012
		Age 90	4.49	1.86	4.61	3.27	0.41	0.38	0.03	1.92	6,224
D5&D6	COVID-19 decedents	All ages	6.63	4.55	5.29	5.90	0.69	0.49	1.26	4.32	7,060
		Age 60	17.30	3.56	18.33	3.86	0.21	0.11	-1.33	4.25	28
		Age 75	9.08	3.29	9.55	5.17	0.36	0.28	-0.45	2.10	210
		Age 85	4.71	2.12	4.47	3.61	0.45	0.39	0.44	2.12	347
		Age 90	3.51	1.68	3.01	2.58	0.48	0.36	0.63	2.39	250
	Everyone else	All ages	16.78	5.70	17.60	8.19	0.34	0.24	-0.54	2.60	1,398,504
		Age 60	19.20	1.86	19.69	1.73	0.10	0.04	-2.59	15.13	46,925
		Age 75	11.91	2.40	12.41	2.80	0.20	0.11	-1.35	5.33	29,366
		Age 85	6.68	2.09	7.03	2.87	0.31	0.21	-0.55	2.60	12,667
		Age 90	4.73	1.83	4.90	3.03	0.39	0.32	-0.14	1.96	5,976
	Other-cause death	All ages	7.59	5.17	6.19	6.95	0.68	0.50	1.04	3.53	52,665
		Age 60	15.47	4.98	17.01	7.11	0.32	0.22	-0.90	2.78	453
		Age 75	8.80	3.73	9.39	6.14	0.42	0.35	-0.29	2.02	1,442
		Age 85	5.12	2.26	5.10	3.84	0.44	0.38	0.16	2.05	2,060
		Age 90	3.73	1.72	3.44	2.79	0.46	0.38	0.47	2.20	1,723
	Population	All ages	16.73	5.74	17.56	8.25	0.34	0.24	-0.54	2.59	1,405,564
		Age 60	19.20	1.86	19.69	1.73	0.10	0.04	-2.60	15.11	46,953
		Age 75	11.89	2.42	12.39	2.82	0.20	0.12	-1.35	5.29	29,576
		Age 85	6.63	2.11	6.99	2.97	0.32	0.22	-0.52	2.53	13,014
		Age 90	4.68	1.84	4.86	3.11	0.39	0.34	-0.11	1.94	6,226
D7&D8	COVID-19 decedents	All ages	6.67	4.74	5.32	6.01	0.71	0.50	1.24	4.16	6,004
		Age 60	16.83	3.72	17.15	3.16	0.22	0.09	-1.19	4.60	20
		Age 75	8.97	3.56	9.91	5.75	0.40	0.33	-0.41	2.01	135
		Age 85	5.01	2.27	4.95	3.80	0.45	0.39	0.23	1.86	258

		Age 90	3.35	1.69	2.71	2.46	0.50	0.35	0.79	2.59	255
	Everyone else	All ages	17.27	5.63	18.32	7.88	0.33	0.22	-0.66	2.77	1,399,562
		Age 60	19.89	1.86	20.09	2.16	0.09	0.05	-2.35	14.77	46,929
		Age 75	12.51	2.33	13.03	2.67	0.19	0.10	-1.52	6.11	29,440
		Age 85	6.94	2.09	7.34	2.85	0.30	0.20	-0.66	2.75	12,755
		Age 90	4.79	1.83	5.02	3.00	0.38	0.32	-0.20	1.95	5,969
	Other-cause death	All ages	7.60	5.22	6.18	7.06	0.69	0.51	1.05	3.52	48,181
		Age 60	15.97	5.55	18.01	7.48	0.35	0.23	-0.92	2.56	368
		Age 75	9.42	3.80	10.08	6.24	0.40	0.33	-0.43	2.06	1,293
		Age 85	5.21	2.32	5.21	3.94	0.45	0.39	0.16	1.96	1,906
		Age 90	3.85	1.77	3.65	3.00	0.46	0.40	0.42	2.06	2,435
	Population	All ages	17.23	5.67	18.30	7.94	0.33	0.22	-0.66	2.76	1,405,566
		Age 60	19.89	1.86	20.09	2.16	0.09	0.05	-2.35	14.77	46,949
		Age 75	12.50	2.35	13.01	2.68	0.19	0.10	-1.52	6.09	29,575
		Age 85	6.90	2.11	7.31	2.88	0.31	0.20	-0.64	2.70	13,013
		Age 90	4.73	1.85	4.96	3.12	0.39	0.34	-0.16	1.92	6,224
D9&D10 (richest)	COVID-19 decedents	All ages	6.31	4.66	4.80	5.57	0.74	0.50	1.41	4.77	4,806
		Age 60	16.34	4.35	17.07	4.43	0.27	0.13	-0.59	2.41	16
		Age 75	8.97	3.83	9.70	6.70	0.43	0.40	-0.24	1.84	108
		Age 85	4.75	2.27	4.41	3.86	0.48	0.40	0.38	1.93	258
		Age 90	3.47	1.66	3.06	2.44	0.48	0.36	0.67	2.46	215
	Everyone else	All ages	18.06	5.98	19.07	8.64	0.33	0.23	-0.64	2.66	1,400,699
		Age 60	21.09	1.73	21.30	1.62	0.08	0.04	-2.99	20.77	46,932
		Age 75	12.88	2.30	13.44	2.56	0.18	0.10	-1.66	6.66	29,466
		Age 85	7.08	2.02	7.50	2.74	0.28	0.19	-0.78	2.96	12,761
		Age 90	4.93	1.77	5.21	2.72	0.36	0.27	-0.37	2.11	6,007
	Other-cause death	All ages	7.61	5.34	6.15	6.78	0.70	0.49	1.15	3.83	43,588
		Age 60	16.98	5.63	19.12	7.43	0.33	0.21	-0.98	2.74	320
		Age 75	9.47	3.92	10.21	6.62	0.41	0.35	-0.38	1.99	1,077
		Age 85	5.40	2.27	5.54	4.07	0.42	0.39	0.01	1.87	1,835
		Age 90	3.88	1.76	3.77	3.03	0.45	0.41	0.31	1.99	1,640
	Population	All ages	18.02	6.02	19.05	8.69	0.33	0.23	-0.64	2.65	1,405,505
		Age 60	21.09	1.74	21.30	1.63	0.08	0.04	-2.99	20.75	46,948
		Age 75	12.87	2.32	13.44	2.58	0.18	0.10	-1.67	6.65	29,574
		Age 85	7.03	2.05	7.47	2.78	0.29	0.19	-0.77	2.89	13,019
		Age 90	4.88	1.79	5.16	2.81	0.37	0.29	-0.34	2.06	6,222

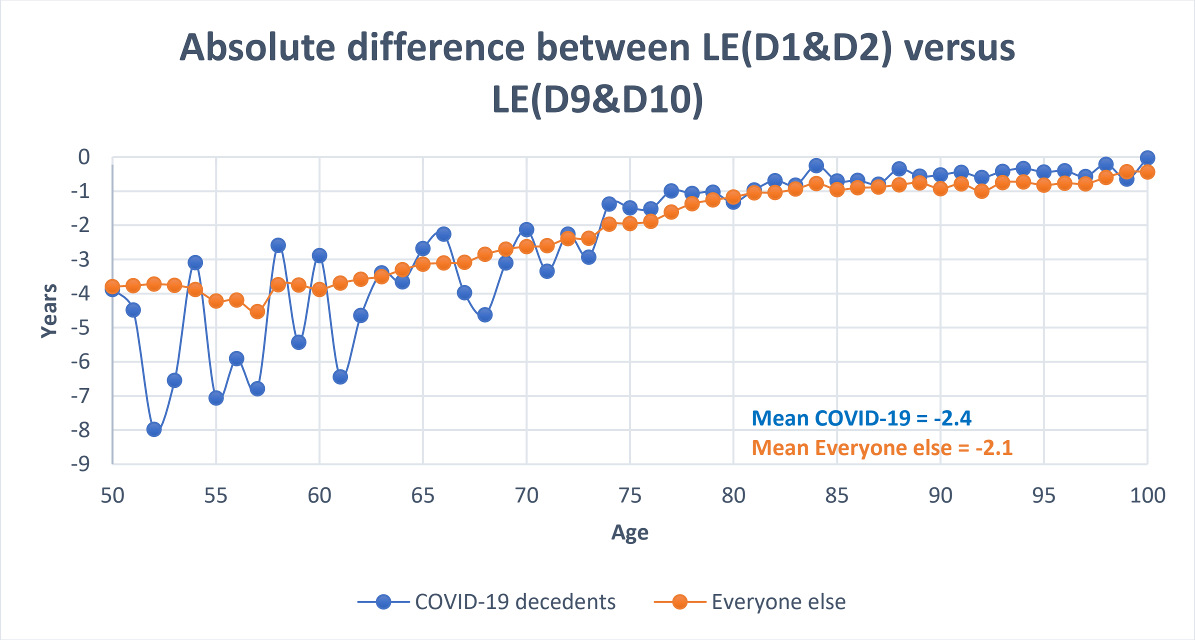

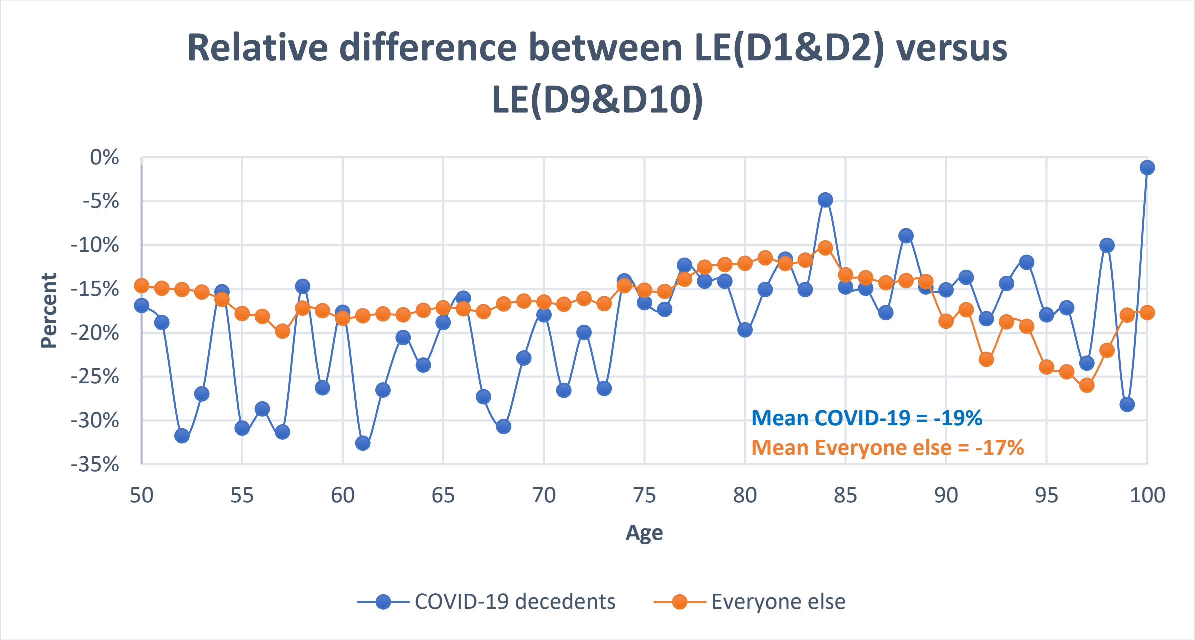

Figure S9 Average absolute and relative difference conditional on age in the life expectancy of the poorest (D1&D2) versus richest (D9&D10) who died of COVID-19 and the rest of the population in the span of 2020-2021. Relative difference was calculated as $\left(\frac{LE^{COVID-19}(D1\&D2,age)}{LE^{COVID-19}(D9\&D10,age)} - 1\right) * 100$

Table S15 COVID-19 deaths, average age-at-death and average years of life lost for the population of the Netherlands who were aged 50+ at the start of 2020 and deceased due to COVID-19 in the span of 2020, by sex and income. Income deciles were clustered into income quintiles, with D1&D2, for example, representing the poorest 20% of the population.

COVID-19 decedents, 2020 only						
				Average years of life lost[5]		
		Number of COVID-19 deaths	Age-at-death[1]	Standard life table[3]	Income-stratified life table[3,4]	Individual-level LE method[2]
Women	D1&D2 (poorest)	3,216 (34.53%)	82.64	8.77 (38%)	7.32 (33%)	5.13 (33%)
	D3&D4	1,924 (20.66%)	83.35	8.37 (21%)	8.26 (22%)	5.77 (22%)
	D5&D6	1,555 (16.7%)	84.32	7.75 (16%)	8.25 (18%)	5.53 (17%)
	D7&D8	1,441 (15.47%)	84.99	7.39 (14%)	7.88 (16%)	5.39 (16%)
	D9&D10 (richest)	1,178 (12.65%)	86.19	6.72 (11%)	7.24 (12%)	5.07 (12%)
	All income groups	9,314 (100%)	83.89	8.04 (100%)	7.74 (100%)	5.36 (100%)
Men	D1&D2 (poorest)	3,373 (31.69%)	78.92	9.52 (34%)	8.35 (31%)	5.92 (31%)
	D3&D4	2,344 (22.02%)	80.1	9.75 (24%)	8.62 (22%)	6.15 (22%)
	D5&D6	1,932 (18.15%)	81.14	8.14 (17%)	8.23 (18%)	6.03 (18%)
	D7&D8	1,628 (15.3%)	81.02	8.3 (14%)	8.68 (16%)	6.44 (16%)
	D9&D10 (richest)	1,367 (12.84%)	81.78	7.77 (11%)	8.56 (13%)	6.21 (13%)
	All income groups	10,644 (100%)	80.28	8.69 (100%)	8.46 (100%)	6.11 (100%)
Both	D1&D2 (poorest)	6,589 (33.01%)	80.74	9.15 (36%)	7.85 (32%)	5.53 (32%)
	D3&D4	4,268 (21.38%)	81.58	8.59 (22%)	8.46 (22%)	5.98 (22%)
	D5&D6	3,487 (17.47%)	82.56	7.97 (17%)	8.24 (18%)	5.81 (18%)
	D7&D8	3,069 (15.38%)	82.88	7.87 (14%)	8.31 (16%)	5.95 (16%)
	D9&D10 (richest)	2,545 (12.75%)	83.82	7.28 (11%)	7.95 (12%)	5.69 (13%)
	All income groups	19,958 (100%)	81.96	8.39 (100%)	8.13 (100%)	5.76 (100%)

1 Age-at-death is as of 1-Jan-20

2 Individual-level LE is as of 1-Jan-20

3 Life tables were calculated based on the 2019 population aged 50+ and stratified by sex.

4 Income was taken as income deciles in the income-stratified life table method

5 Values in brackets are percentage of the total YLL burden by sex group.

Table S16 COVID-19 deaths, average age-at-death and average years of life lost for the population of the Netherlands who were aged 50+ at the start of 2020 and deceased due to COVID-19 in the span of 2021, by sex and income quintile. Income deciles were clustered into income quintiles, with D1&D2, for example, representing the poorest 20% of the population.

COVID-19 decedents, 2021 only						
				Average years of life lost[5]		
		Number of COVID-19 deaths	Age-at-death[1]	Standard life table[3]	Income-stratified life table[3,4]	Individual-level LE method[2]
Women	D1&D2 (poorest)	2,877 (33.03%)	81.09	9.82 (37%)	8.29 (32%)	6.56 (33%)
	D3&D4	1,911 (21.94%)	82.03	9.24 (23%)	9.17 (24%)	6.90 (23%)
	D5&D6	1,591 (18.27%)	83.51	8.32 (17%)	8.83 (19%)	6.57 (18%)
	D7&D8	1,286 (14.76%)	84.44	7.83 (13%)	8.34 (14%)	6.39 (14%)
	D9&D10 (richest)	1,045 (12%)	85.51	7.12 (10%)	7.69 (11%)	5.85 (11%)
	All income groups	8,710 (100%)	82.77	8.8 (100%)	8.51 (100%)	6.52 (100%)
Men	D1&D2 (poorest)	3,312 (31.26%)	77.94	10.15 (34%)	8.91 (31%)	7.19 (31%)
	D3&D4	2,436 (22.99%)	79.34	9.28 (23%)	9.12 (23%)	7.23 (23%)
	D5&D6	1,982 (18.71%)	79.97	8.89 (18%)	8.98 (19%)	7.33 (19%)
	D7&D8	1,649 (15.56%)	80.6	8.56 (14%)	8.96 (16%)	7.45 (16%)
	D9&D10 (richest)	1,216 (11.48%)	81.22	8.21 (10%)	9.05 (12%)	7.19 (11%)
	All income groups	10,595 (100%)	79.43	9.24 (100%)	8.99 (100%)	7.27 (100%)
Both	D1&D2 (poorest)	6,189 (32.06%)	79.4	9.99 (35%)	8.62 (31%)	6.90 (32%)
	D3&D4	4,347 (22.52%)	80.52	9.26 (23%)	9.12 (23%)	7.08 (23%)
	D5&D6	3,573 (18.51%)	81.55	8.63 (18%)	8.92 (19%)	6.99 (19%)
	D7&D8	2,935 (15.2%)	82.28	8.24 (14%)	8.69 (15%)	6.99 (15%)
	D9&D10 (richest)	2,261 (11.71%)	83.2	7.7 (10%)	8.42 (11%)	6.57 (11%)
	All income groups	19,305 (100%)	80.94	9.04 (100%)	8.77 (100%)	6.93 (100%)

1 Age-at-death is as of 1-Jan-21

2 Individual-level LE is as of 1-Jan-21

3 Life tables were calculated based on the 2019 population aged 50+ and stratified by sex.

4 Income was taken as income deciles in the income-stratified life table method

5 Values in brackets are percentage of the total YLL burden by sex group.

Table S17 COVID-19 deaths, average age-at-death and average years of life lost for the population of the Netherlands who were aged 50+ at the start of 2020 and deceased due to COVID-19 in the span of 2020-2021, by sex and income quintile, by sex and income quintile. Income deciles were clustered into income quintiles, with D1&D2, for example, representing the poorest 20% of the population.

COVID-19 decedents, 2020-2021 combined						
				Average years of life lost[5]		
		Number of COVID-19 deaths	Age-at-death[1]	Individual-level LE method[2]	Standard life table[3]	Income-stratified life table[3,4]
Women	D1&D2 (poorest)	6,093 (33.8%)	81.44	5.98 (33%)	9.54 (37%)	8.01 (32%)
	D3&D4	3,835 (21.28%)	82.20	6.56 (23%)	9.08 (22%)	8.96 (23%)
	D5&D6	3,146 (17.45%)	83.41	6.28 (18%)	8.31 (17%)	8.82 (18%)
	D7&D8	2,727 (15.13%)	84.26	6.07 (15%)	7.84 (14%)	8.35 (15%)
	D9&D10 (richest)	2,223 (12.33%)	85.40	5.64 (11%)	7.13 (10%)	7.71 (11%)
	All income groups	18,024 (100%)	82.86	6.13 (100%)	8.67 (100%)	8.37 (100%)
Men	D1&D2 (poorest)	6,685 (31.48%)	77.94	6.75 (31%)	10.13 (35%)	8.88 (31%)
	D3&D4	4,780 (22.51%)	79.21	6.92 (23%)	9.31 (23%)	9.15 (23%)
	D5&D6	3,914 (18.43%)	80.04	6.91 (18%)	8.8 (18%)	8.89 (18%)
	D7&D8	3,277 (15.43%)	80.31	7.18 (16%)	8.7 (15%)	9.1 (16%)
	D9&D10 (richest)	2,583 (12.16%)	81.04	6.88 (12%)	8.22 (11%)	9.06 (12%)
	All income groups	21,239 (100%)	79.36	6.9 (100%)	9.24 (100%)	8.99 (100%)

1 Age-at-death is as of 1-Jan-20

2 Individual-level LE is as of 1-Jan-20 for all decedents

3 Income was taken as income deciles and 2019 population 50+ data stratified by sex was used for the life table

4 Values in brackets are percentage of the total YLL burden by sex group.

Table S18 ANOVA disaggregation of the life expectancy variation.

ANOVA	Sum of squares	Deg. of freedom	Percentage of total variance
Variance disaggregation by income quintile			
Between income groups	32.21	4	0.21%
Within income groups	20,220.22	39,258	99.79%

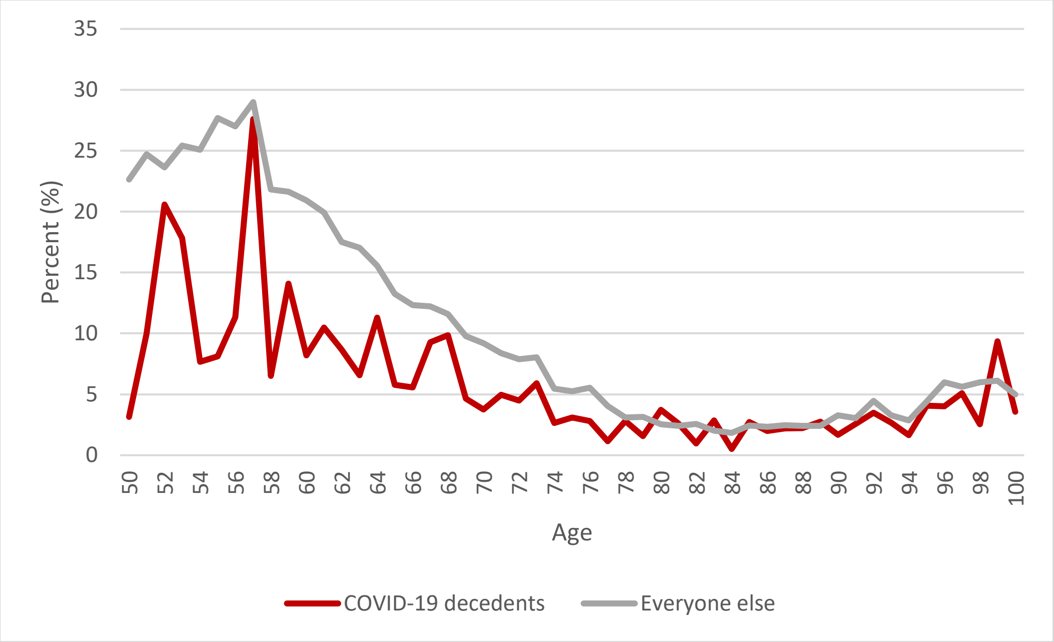

Figure S10 Percent of the age-specific variance in life expectancy that is explained by income (defined as income quintiles).

References

Breslow, N. E. (1972). Discussion on Professor Cox's Paper. *Journal of the Royal Statistical Society: Series B (Methodological)*, *34*(2), 202–220. https://doi.org/10.1111/J.2517-6161.1972.TB00900.X

Deryugina, T., Heutel, G., Miller, N. H., Molitor, D., & Reif, J. (2019). The Mortality and Medical Costs of Air Pollution: Evidence from Changes in Wind Direction. *American Economic Review*, *109*(12), 4178–4219. https://doi.org/10.1257/AER.20180279

Eurostat/OECD/WHO IHMT. (n.d.). Retrieved June 27, 2023, from https://stats.oecd.org/health_questionnaire/ishmt/jqnmhc_ishmt.pdf

James, G., Witten, D., Hastie, T., & Tibshirani, R. (2021). *An Introduction to Statistical Learning with Applications in R Second Edition*.

Newson, R. B. (2010). Comparing the predictive powers of survival models using Harrell's C or Somers' D. *The Stata Journal*, *10*(3), 339–358.

Parner, E. T., & Andersen, P. K. (2010). Regression analysis of censored data using pseudo-observations. *The Stata Journal*, *10*(3), 408–422.

Royston, P. (2014). Tools for checking calibration of a Cox model in external validation: Approach based on individual event probabilities. *The Stata Journal*, *14*(4), 738–755.

Siermann, C., van Teeffelen, P., & Urlings, L. (2004). Equivalence factors 1995-2000. *Statistics Netherlands (Centraal Bureau Voor de Statistiek)*. https://www.cbs.nl/nl-nl/achtergrond/2004/25/equivalentiefactoren-1995-2000

Uno, H., Cai, T., Pencina, M. J., D'Agostino, R. B., & Wei, L. J. (2011). On the C-statistics for Evaluating Overall Adequacy of Risk Prediction Procedures with Censored Survival Data. *Statistics in Medicine*, *30*(10), 1105. https://doi.org/10.1002/SIM.4154
